# The full spectrum of ethical issues in pediatric genome-wide sequencing: a systematic qualitative review

**DOI:** 10.1186/s12887-021-02830-w

**Published:** 2021-09-06

**Authors:** Johanna Eichinger, Bernice S. Elger, Insa Koné, Isabel Filges, David Shaw, Bettina Zimmermann, Stuart McLennan

**Affiliations:** 1 Institute for Biomedical Ethics, University of Basel, Bernoullistrasse 28, 4056 Basel, Switzerland; 2 Institute of History and Ethics in Medicine, Technical University of Munich, Munich, Germany; 3 Center for legal medicine (CURML), University of Geneva, Geneva, Switzerland; 4 Medical Genetics, Institute of Medical Genetics and Pathology, University Hospital Basel and University of Basel, Basel, Switzerland; 5 Department of Clinical Research, University Hospital Basel and University of Basel, Basel, Switzerland; 6 Care and Public Health Research Institute, Maastricht University, Maastricht, The Netherlands

**Keywords:** Whole genome sequencing, Whole exome sequencing, Genome-wide sequencing, Pediatrics, Children, Ethical, legal and social issues

## Abstract

**Background:**

The use of genome-wide sequencing in pediatric medicine and research is growing exponentially. While this has many potential benefits, the normative and empirical literature has highlighted various ethical issues. There have not been, however, any systematic reviews of these issues. The aim of this systematic review is to determine systematically the spectrum of ethical issues that is raised for stakeholders in in pediatric genome-wide sequencing.

**Methods:**

A systematic review in PubMed and Google Books (publications in English or German between 2004 and 2021) was conducted. Further references were identified via reference screening. Data were analyzed and synthesized using qualitative content analysis. Ethical issues were defined as arising when a relevant normative principle is not adequately considered or when two principles come into conflict.

**Results:**

Our literature search retrieved 3175 publications of which 143 were included in the analysis. Together these mentioned 106 ethical issues in pediatric genome-wide sequencing, categorized into five themes along the pediatric genome-wide sequencing lifecycle. Most ethical issues identified in relation to genome-wide sequencing typically reflect ethical issues that arise in general genetic testing, but they are often amplified by the increased quantity of data obtained, and associated uncertainties. The most frequently discussed ethical aspects concern the issue of unsolicited findings.

**Conclusion:**

Concentration of the debate on unsolicited findings risks overlooking other ethical challenges. An overarching difficulty presents the terminological confusion: both with regard to both the test procedure/ the scope of analysis, as well as with the topic of unsolicited findings. It is important that the genetics and ethics communities together with other medical professions involved work jointly on specific case related guidelines to grant the maximum benefit for the care of the children, while preventing patient harm and disproportionate overload of clinicians and the healthcare system by the wealth of available options and economic incentives to increase testing.

**Supplementary Information:**

The online version contains supplementary material available at 10.1186/s12887-021-02830-w.

## Background

Genome-wide sequencing, as whole exome or whole genome sequencing (WGS/ WES), can be used to identify variations in a person’s genetic code that might lead to impaired development and disease or disability, that might be ‘otherwise undetectable through clinical history, physical examination, and biochemical or metabolic tests’ [1]. With genome-wide sequencing becoming increasingly faster and more affordable, it is expected that it will have an enormous impact on scientific research, clinical practice, and wider society [2–4]. The use of genome-wide sequencing in pediatrics is particularly growing exponentially, and it is hoped that it will help children with undiagnosed genetic diseases to end their diagnostic odyssey sooner and cheaper [5–8]. The diagnostic and clinical utility of WGS and WES in children with suspected monogenic disorders has been demonstrated in various studies [9–11]. Projects such as the Deciphering Developmental Disorders study, offering exome sequencing to children with severe developmental disorders, estimates that if a clinical exome was offered as a first line diagnostic test to children and their parents, over half of these children would instantly receive a diagnosis [12, 13].

In addition to the potential benefits of genome-wide sequencing in pediatrics, however, the empirical and normative literature has also highlighted a number of important regulatory and ethical challenges [14–24]. These ethical issues are often even more challenging in the context of genome-wide sequencing in children, as parents then make decisions for them: complex issues around the child’s future autonomy, parental autonomy, the best interests of the patient, and also the best interests of the wider family have to be considered [21, 25, 26]. Decision-making here needs to integrate not only concern for the long-term welfare of a child or young person, or possible future children, but also for other members of the family. In biomedical ethics, ethical challenges are commonly evaluated using the four Principles of Biomedical Ethics [27]: Beneficence, nonmaleficence, respect for autonomy, and justice.

To date, however, there have not been any systematic reviews of these ethical challenges. Although there have been previous review articles conducted on ethical issues in genome-wide sequencing [2, 28–30], these have been either narrative (non-systematic) reviews or limited in scope due to their focus on a few particular issues or on empirical research only.

With the increasing use of genome-wide sequencing, non-genetic medical specialties, such as pediatricians, are also increasingly confronted with it: They are often the first to see and know the affected patient and their families best; they provide pre- and post-test care; they are able to make a referral to a geneticist or, in some countries, can order genome-wide sequencing of their pediatric patients themselves. It is important that these health care practitioners have a comprehensive overview of ethical issues that may arise to guide their decision-making. This systematic qualitative review aims to determine systematically the spectrum of ethical issues that is raised for stakeholders in in pediatric genome-wide sequencing.

## Methods

The methods of the study are presented in accordance with the Preferred Reporting Items for Systematic Reviews and Meta-Analysis (PRISMA) as far as they are applicable to qualitative analysis [31].

### Inclusion criteria

To be included, publications had to describe and/or assess an ethical issue involved in genome-wide sequencing with children via either conceptual or empirical methods. The definition of ethical issues was based on principlism [27] which has been successfully used in other systematic qualitative ethics reviews [32–35]. It was assumed that an ethical issue arises when 1) one or more principles have been neglected, or 2) because of conflicts between two or more ethical principles. Genome-wide sequencing was considered to include the terms (whole) genome sequencing, (whole) exome sequencing, genomic sequencing, genome-wide sequencing, genome scale sequencing or complete genome sequencing.

Due to the composition of our research team, only publications in English or German were included*.* Furthermore, publications needed to be a journal article, book or book chapter, or a national-level report published from April 14, 2003 to May 28, 2021. The date limit was added at the last step of the PubMed search, all other inclusion criteria were applied when screening Google Books and PubMed results. Publications before that date were excluded, because the Human Genome Project had not been completed [36]. The methodological quality, beyond the fact that the paper was identified in scientific databases and published in peer-reviewed journals, did not serve as a criterion of eligibility criteria, as the quality of a publication was irrelevant for the purpose of identifying the spectrum of ethical issues.

### Search strategy and data sources

The search terms were developed through an iterative process, where combinations of key words and MeSH terms were piloted in PubMed and the results were assessed for inclusion of a known set of representative literature. This resulting combination of key words and MeSH terms included in the search strategy are presented in Table 1. The search was conducted on May 28, 2021. Google Books was also searched with the search strings “whole genome sequencing” AND ethics as well as “whole exome sequencing” AND ethics. Due to the large number of hits and because Google Books sorts hits by relevance, only the first 100 publications were included. Further publications were identified by screening the reference lists of the included publications.

**Table 1:** Search Strategy in PubMed

Ethics	1	Ethics [MeSH Terms]	149,276
	2	ethic*[Title/Abstract]	144,284
	3	1 OR 2	236,299
Specific Ethical Issues	4	Personal Autonomy [MeSH Terms] OR Informed Consent [MeSH Terms] OR Confidentiality [MeSH Terms] OR Privacy [MeSH Terms]	110,138
	5	autonomy [Title/Abstract] OR consent [Title/Abstract] OR confidentiality [Title/Abstract] OR privacy [Title/Abstract] OR incidental finding*[Title/Abstract] OR variant* of unknown significance [Title/Abstract] OR secondary finding*[Title/Abstract]	130,365
	6	4 OR 5	205384
	7	3 OR 6	385419
Genome-wide sequencing	8	Whole genome sequencing [MeSH Terms]] OR Genomics [MeSH Terms]] OR Sequence Analysis, DNA [MeSH Terms]	351684
	9	whole-genome sequencing [Title/Abstract] OR whole-exome sequencing [Title/Abstract] OR genome sequencing [Title/Abstract] OR exome sequencing [Title/Abstract] OR genomic sequencing [Title/Abstract] OR genomic test*[Title/Abstract] OR genomic stud*[Title/Abstract] OR complete genome sequencing [Title/Abstract] OR genome-scale sequencing [Title/Abstract]	53,146
	10	8 OR 9	385161
	11	7 AND 10	3894
		With Filter from 14.4.2003 until 28.5.2021	3070

### Study selection

Based on the inclusion criteria, JE along with either SM, DS or BZ independently screened all titles and abstracts in order to assess their eligibility for inclusion for full text screening to insure inter-rater validity. Furthermore, JE and SM screened the back cover descriptions and tables of content of the Google Book’s hits and excluded those not containing any relevant chapters. In case of disagreement, consensus was reached discursively. Full texts of potentially eligible studies were then screened by JE again along with either SM or IK. Excel sheets were used for the entire screening process. Any discrepancies between reviewers during the screening process with regard to the inclusion/exclusion of articles was resolved by consensus.

### Data analysis and synthesis

Included full texts were analyzed using conventional qualitative content analysis [37]. Findings were presented as higher- and lower-level categories in a coding frame, which was developed inductively from the data. Only the highest-level codes were generated deductively for a life-cycle perspective; it was assumed that pediatric genome-wide sequencing has five broad phases: (1) the decision regarding when to use genome-wide sequencing, (2) pretest counselling, (3) sequencing, analysis and interpretation, (4) communicating results, and (5) future use of data. JE and SM read and coded five articles purposefully selected to identify inductively as many ethical issues as possible. JE compared the extracted quotes and paraphrases across reviewers and publications and constructed a preliminary coding framework using the qualitative data analysis software MAXQDA. The draft framework was discussed during regular meetings with the research team to increase validity and reliability. For the next five publications, JE and SM again extracted relevant quotes, checking whether the existing coding framework already described the relevant issues, and introduced new categories where necessary. JE integrated the findings and the results were constantly discussed among SM and JE. The remaining publications were analyzed by JE, applying the defined categories and introducing new ones if necessary. Further in-person meetings with co-authors were convened to help resolve any remaining coding problems, and to discuss the framework’s consistency and comprehensibility until all authors agree upon the final matrix of ethical issues.

## Results

**Fig. 1:**
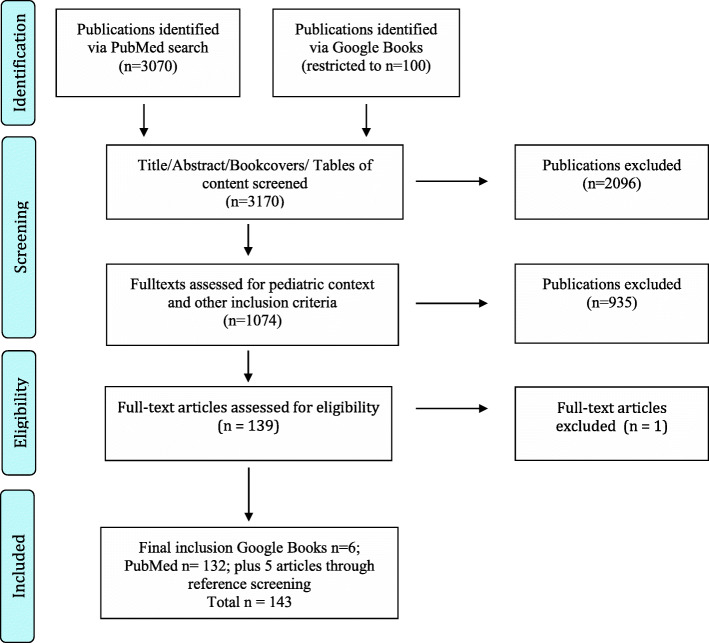
Flow chart illustrating the systematic article selection process

The literature search identified 3175 publications of which 143 were included in the final analysis (see Fig. 1). Of these, 96% (*n* = 137) were journal articles and the remaining (*n* = 6) were book chapters. The vast majority of included publications were published after 2014. A list providing full bibliographical information of all 143 publications is provided in Additional file 1.

### Ethical issues

A total of 106 distinct ethical issues in the application of genome-wide sequencing in a pediatric population were identified (Table 2). The main findings categorized according to the different phases of the genome-wide sequencing lifecycle include:

- *Issues related to when to use genome-wide sequencing:* These ethical issues relate to the questions, if and for which indications pediatric genome-wide sequencing should be used, what the potential risks associated with it are, and the general challenges for the involved clinicians and researchers. For example, the risks of extended newborn screening with WGS/WES, the risks of lacking expertise, training and time especially of non-genetics specialist involved in decision-making, and the risk of injustice due to unequal access to reimbursement by insurances.
- *Issues related to pretest counselling:* These ethical issues relate to the general challenges for the informed consent process; including what should be discussed during pretest counselling (e.g. the potential for results to change over time, the potential effects on parent/child bonding), whether there should be different forms of consent and directiveness in counselling depending on the urgency of the situation, the challenges of parental decision-making on behalf of their child, and the challenges to decide how much choice parents should have regarding what types of findings are received.
- *Issues related to sequencing, analysis and interpretation:* Here the ethical issues include challenges regarding the infrastructure, such as the risk of inconsistencies and variability due to different bioinformatics pipelines; challenges regarding the interpretation of variants due to the gap between the amount of data which are generated and the knowledge to use them in a clinical context; and the challenge to decide whether it should be actively searched for a certain list of disease-associated genes in every genome-wide sequencing.
- *Issues related to communicating results:* The main ethical issues repeatedly raised here relate to the challenge of reporting unsolicited findings (UFs), e.g. the risk of undermining the parents/participants/patients right not to know with an obligatory disclosure of certain UFs; the challenge of balancing the best interests of the child with the best interests of the family regarding the disclosure of UFs; the risk of the diagnosis negatively impacting the parent-child bonding; and the challenges of thoughtfully and effectively framing the results.
- *Issues related to future use of data:* These ethical relate mostly to the challenges of data sharing, storage and governance, such as the challenge to determine whose responsibility it is to initiate reanalysis, provide access and recontact patients/parents/participants (especially when pediatric patients reach majority), the risk to privacy and the risk of risk of genetic discrimination (insurance, labor market, access to future medical intervention).

**Table 2:** gives a full and detailed account of issues identified. The full spectrum of ethical issues in genome-wide sequencing coding framework with example quotations

THEME			
CODE	SUBCODE	PUBLICATIONS	EXAMPLE QUOTE
ISSUES RELATED TO WHEN TO USE GENOME-WIDE SEQUENCING			
Challenges in deciding who should be tested	Risks of Direct-to-consumer Testing	Bunnik, et al. 2013 Borry, et al. 2014 Howard, et al. 2015 Joseph, et al. 2016 Sabatello and Appelbaum 2016 Johnston, et al. 2018 Zacharias, et al. 2018 Benedetti and Marron 2021	Accordingly, the aim of DTC testing has shifted from the prediction of an individual’s genetic risk for a single disease to something like ‘getting to know as much as possible’ on the basis of a genome-wide scan. […] The standards of pre-test information provision and informed consent used in clinical genetics can hardly be met in a relatively under- regulated commercial context, in which professional knowledge, skills and values are often lacking. Lack of adequate information and informed consent may harm consumers both directly and indirectly: directly through the receipt of unwanted and potentially harmful information [e.g. knowing that one is at increased risk for an untreatable or unpreventable disease, such as Alzheimer’s disease], and indirectly through misunderstanding or misinterpretation, and associated personal, social and health risks. […] Informed consent is needed not only to help prevent the potential harms associated with personal genome testing, but also to help ensure that genetic testing is the result of an autonomous decision rather than the ‘inconsiderate’ acceptance of a commercial offer [38].
	Risks of predictive testinggenerally	ACMG 2013 Editorial 2013 Borry, et al. 2014 Knoppers, Avard, et al. 2014 Berg and Powell 2015 Botkin, et al. 2015 Char 2015(a) Hens and Dierickx 2015 Levenson 2015 Sabatello and Appelbaum 2015 Bowdin et al. 2016 Hufnagel, et al. 2016 Sabatello and Appelbaum 2016 Johnson, et al. 2017 Casas 2018 Johnston, et al. 2018 Garrett, et al. 2019 Johnson, et al. 2019 Lantos 2019(a) Szego, et al. 2019 Hay, et al. 2021	The family that has a baby’s genome sequenced just to see what might be found may spend years worrying about that cancer risk in their perfectly healthy child [39].
	Risks of extending newborn screening with WGS/WES	Goldenberg and Sharp 2012 Tarini and Goldenberg 2012 Bunnik, et al. 2013 Knoppers, Sénécal, et al. 2014 Landau, et al. 2014 Berg and Powell 2015 Botkin, et al. 2015 Howard, et al. 2015 Reinstein 2015 Botkin and Rothwell 2016 Joseph, et al. 2016 King and Smith 2016 Lantos 2016 Friedman, et al. 2017 Iskrov, et al. 2017 Seidel 2017 Yang, et al. 2017 Johnston, et al. 2018 Zacharias, et al. 2018 Boardman, et al. 2019 Genetti, et al. 2019 Rothwell and Botkin 2019 Chaudhari, et al. 2020 Downie, et al. 2020 Moultrie, et al. 2020 Wolf, et al. 2020 Cabello, et al. 2021 Levy 2021 Newson 2021	All of the ethical and public policy issues associated with current newborn screening practices apply to genome- wide sequencing as well, and many of these issues are exacerbated by the fact that genome-wide sequencing produces much more information about the individual than conventional testing does. For example, it is more difficult (or impossible) to justify mandatory screening, even if families have the ability to opt out, if many additional screening targets are added, especially if the benefits of screening for some of these additional targets are uncertain. At the very least, genomic newborn screening would require ensuring that parents have sufficient, clearly-understandable information available about the screening program and that the entire population has access to confirmatory diagnostic and treatment services, including genetic counselling. Maintaining effective governance and efficient administration of population-based genomic newborn screening programs would also be essential to avoid losing the high participation rates and widespread public support that these programs currently enjoy [40].
	Challenge to deal with parental requests to test	May 2013 Sabatello and Appelbaum 2016	1 author (TM) has seen the effect of pleas from parents for access to this new, and available, diagnostic technology. As we have discussed elsewhere, such pleas are often discounted in health care policy as emotion-based and therefore less worthy of recognition as legitimate motives to go forward with intervention (although often readily accepted as reasons to refrain). However, there are legitimate reasons to recognize the motivational force of emotions felt by parents when confronted by the uncertainties that accompany a confounding debilitating disease suffered by their children, and the cycle of testing and re-testing inflicted on the children for whom diagnosis, and therefore settled treatment approach, has eluded attending physicians [41].
	Challenge to deal with minor’s request to test without parental permission	Clayton 2015 Sabatello and Appelbaum 2016	The simple answer is that unemancipated minors have virtually no legal rights to obtain genetic or genomic testing without parental permission [42].
	Challenge to decide whether and under which conditions children should participate in genomic research	Wilfond and Diekema 2012 Knoppers, Avard, et al. 2014 Rahimzadeh 2017 Sundby, et al. 2018	Research with children, and indeed with situationally vulnerable groups generally, therefore raise an ethical tension. Children warrant special ethical protections as a result of their situational vulnerability. They should not, however, be categorically excluded from research that anticipates the contribution of new knowledge that could improve their health and well-being. Although this tension is not new, the types of risks genomic data sharing poses to children and the approaches research ethics review committees employ to minimize them are unique [43].
	Challenge to decide who should get priority	Gyngell, et al. 2019	In the absence of sufficient capacity to offer RGT for all infants in the NICU who may potentially benefit, there will be a need to prioritize. It is likely that in the early phase at least, RGT will be restricted to those infants with clinical features that are highly suggestive of an underlying genetic condition. […] An alternative approach would be to prioritize infants where the result of RGT is expected to be of high clinical utility, for example where a diagnosis would potentially help parents considering treatment limitation decisions, an expensive intervention such as transplantation, or in cases where parents are considering adoption. These are the ‘weightiest’ choices parents can make, and they should have access to useful information to help inform those decisions [44].
	Challenge to decide whether to use as first tier test or after more limited genetic testing	Botkin, et al. 2015 Levenson 2015 Newson and Schonstein 2016 Rogers and Zhang 2016 Bertier, et al. 2017 Szego, et al. 2019	ASHG recommends that, in the context of diagnostic testing for a child with a most likely genetic disorder, genome-scale sequencing is appropriate when prior, more limited genetic testing failed to identify a causative mutation. Depending on the clinical presentation and on the quality and availability of appropriate targeted testing, comprehensive testing such as genome-scale sequencing might also be indicated in certain circumstances, even in the absence of prior, more limited genetic testing [45].
	Challenge to decide whether to do only child or trio testing	Char 2015(b) Bertier, et al. 2017 Casas 2018 Eno, et al. 2018 Vears, et a. 2018 Cornelis and Wouters 2019	There also was debate as to whether just the proband, or the affected child and both parents (trio analysis), should be sequenced. Although the production of sequencing data for trios is three times as expensive, it allows rapid identification of de novo mutations in the proband. Therefore, this approach may be particularly valuable in cases where there is a strong suspicion that the causal mutation appeared de novo in the affected child, or that it has a recessive mode of inheritance [28].
Challenges for decision-making	Risk of lack of experts	Beale, et al. 2015 Johnston, et al. 2018 Graf, et al. 2019 Szego, et al. 2019	The main resource-related issues pertaining to service provision are the need for additional computing capacity, more bioinformaticians, more genetic counsellors and also genetics-related training for the public and a wide range of staff. It is also considered that, as the number of children undergoing genetic testing increases, there will be an increase in demand for information and support for families [46].
	Challenges of cross-disciplinary collaboration	ACMG 2013 Burke 2015 Burke and Clarke 2016 Johnson, et al. 2017 Diamonstein 2019 Gyngell, et al. 2019 Szego, et al. 2019 Chaudhari, et al. 2020 Deuitch, et al. 2020	Caring for children and families who have genetic differences requires a partnership between the primary care pediatrician and the appropriate specialists. Undertaking WES/WGS testing also requires a partnership between pediatricians and genetic specialists until the nuances of genomic testing become better understood by the majority of pediatricians [47].
	Risk of lacking expertise, training and time	ACMG 2013 Knoppers, Sénécal, et al. 2014 Beale, et al. 2015 Botkin, et al. 2015 Burke 2015 Howard, et al. 2015 Bowdin, et al. 2016 Burke and Clarke 2016 Green, et al. 2016 Lantos 2016 Bertier, et al. 2017 Iskrov, et al. 2017 Graf, et al. 2019 Szego, et al. 2019 Byrjalsen, et al. 2020 Deuitch, et al. 2020 Odgis, et al. 2021	In addition to understanding the ethical framework for the disclosure of genomic testing results, pediatricians must have sufficient knowledge of the testing procedures themselves. In the midst of the ethical decisions that must be made in terms of genome sequencing in children is the question regarding the comfort level of the pediatricians in discussing complex genetic test results and testing procedures [47].
	Challenge of high responsibility for clinicians/researchers	May, et al. 2013 Knoppers, Sénécal, et al. 2014 Burke 2015 Friedman, et al. 2019 Gyngell, et al. 2019 Ross and Clayton 2019 Byrjalsen, et al. 2020	There is emerging evidence that healthcare providers who must make high-stakes irrevocable treatment decisions involving genomic results are already experiencing moral distress. There is therefore a need to articulate practical procedures, underpinned by consistent normative principles and values, to help clinicians decide […] [44].
	Challenge to assess clinical value and personal utility of genome-wide sequencing	Beale, et al. 2015 Howard, et al. 2015 Botkin 2016 Bowdin, et al. 2016 Bertier, et al. 2017 Friedman, et al. 2017 Chassagne, et al. 2019 Friedman, et al. 2019 Lantos 2019(a) Malek, et al. 2019	It has been argued that the clinical utility of a genetic test should also include consideration of ethical, legal, and social issues related to the diagnosis, prevention, or treatment of the disease that is being tested. Even this broad definition of clinical utility may not be fully inclusive of the overall costs and benefits of genetic testing: elements of “personal utility” may also need to be considered. As a practical matter, however, personal utility and social consequences are difficult to measure and have contributed little to the funding decisions healthcare systems and insurers have made regarding genetic testing to date [48].
	Difficulty to determine best interests principle	Bush 2014 Holm 2014 Zawati, et al. 2014 Anderson, et al. 2015 Kesserwan, et al. 2016 Newson and Schonstein 2016 Sabatello and Appelbaum 2016 Johnson, et al. 2017 Newson 2017 Friedman, et al. 2019	The central ethical tenet of clinical pediatrics is that the best interests of the child are paramount, but determining the best interests of a severely ill infant may be challenging. For example, some of the benefits attributed to diagnostic GWS result from avoidance of high-intensity treatment and risky medical or surgical interventions in favor of palliative “comfort care” for infants who have uncontrollable suffering or whose prognosis is dismal. Is it in a baby’s best interests for his parents to find out that he has an untreatable genetic condition that has been fatal within the first few months of life in all previously reported cases? [48]
	Risk of rising physical burdens due to increased testing	Tarini and Goldenberg 2012 Bunnik, et al. 2013 Howard, et al. 2015 Lantos 2016 Wouters, et al. 2017 Horton and Lucassen 2019 Lantos 2019(a) Lantos 2019(b) Sachdev, et al. 2021	Ackerman et al.‍ reported a case in which the possibility of inappropriate treatment is illustrated. Doctors tested a first-degree relative of a patient who died of sudden cardiac death. The relative had a genetic finding that was interpreted as likely pathogenic for long QT syndrome (LQTS). The man had no signs or symptoms of LQTS at the time of the molecular diagnosis. Nevertheless, on the basis of that genomic result, the doctor recommended, and the patient received, an implantable defibrillator. The authors criticized the decision and warned that, “The mere presence of a rare variant in a bona fide LQTS- susceptibility gene should not compel a pathogenic, probably deleterious variant rendering.” We do not know how common such situations are, but we do know that interpretations of the likelihood that a particular variant will be classified as pathogenic are constantly changing.‍ Such findings will always create uncertainty among both doctors and patients [49].
	Risk of rising psychological burdens due to increased testing	Goldenberg and Sharp 2012 Tarini and Goldenberg 2012 Abdul-Karim, et al. 2013 Bunnik, et al. 2013 Editorial 2013 Dimmock and Bick, 2014 Knoppers, Avard, et al. 2014 Knoppers, Sénécal, et al. 2014 Allain 2015 Clayton 2015 Hens and Dierickx 2015 Howard, et al. 2015 Reinstein 2015 Bowdin, et al. 2016 Lantos 2016 Newson 2017 Wouters, et al. 2017 Johnston, et al. 2018 Friedman, et al. 2019 Lantos 2019(a) Lantos 2019(b) Robinson, et al. 2019Szego, et al. 2019 Savatt, et al. 2020	There are also potential psychological harms such as alteration of self-image, distortion of parental perception of the child, increased anxiety and guilt, familial stress related to the identification of other at-risk family members, difficulty obtaining life and/or disability insurance, and the detection of non-paternity [50].
Risks of injustice	Challenge of fair distribution of resources in healthcare system	Goldenberg and Sharp 2012 Editorial 2013 Dimmock and Bick, 2014 Howard, et al. 2015 Bowdin, et al. 2016 Lantos 2016 Rogers and Zhang 2016 Wouters, et al. 2017 Johnston, et al. 2018 Chassagne, et al. 2019 Gyngell, et al. 2019 Hart, et al. 2019 Lantos 2019(a) Szego, et al. 2019Cabello, et al. 2021 Newson 2021	Although genetic services and screening programmes aim to improve the health of the population, there is growing concern that the increasing number of genetic tests becoming available at lower costs could compromise the viability of the healthcare system. Even though the tests themselves may be inexpensive and suitable for large-scale use, the infrastructure and human resources needed to provide appropriate education, counseling, interventions and follow-up are likely to be far more costly. When it comes to the allocation of scarce resources, economic considerations must be considered alongside ‘notions of justice, equity, personal freedom, political feasibility, and the constraints of current law [51].
	Risk of unequal access to genome-wide sequencing	May, et al. 2013 Green, et al. 2016 Casas 2018 Grebe, et al. 2020 Cabello, et al. 2021 Odgis, et al. 2021	Chief among these are moral concerns about justice, disparities in access to both testing and intervention, and the differing risks and benefits that may result given different socioeconomic status or racial background. Indeed, the report accompanying the joint AAP/ACMG Policy Statement suggests less actual harm from testing than anticipated but also notes that the little evidence assembled dispropotionately reflects white individuals of higher socioeconomic status. This itself is likely a reflection of disparities in access to new health care technologies. Far more effort is needed, then, to ensure that the significant potential benefits of WGS are fairly distributed and that risks are assessed through consideration of disparate circumstances and resources [52].
	Lack of formal health technology assessments comparing the cost-effectiveness to alternative approaches	Beale, et al. 2015 Bowdin, et al. 2016 Gyngell, et al. 2019	However, despite calls for research focusing on the comparative downstream costs and clinical practice implications of WES/WGS, empiric research is limited. We currently lack formal health technology assessments comparing the cost-effectiveness of WGS to alternative approaches, a major evidence gap that is only just beginning to be rectified. Due to the continual decline in the laboratory costs of sequencing we are approaching the fabled ‘US$1000 genome’. However these are only the incremental laboratory costs for a high-throughput sequencing facility, they do not include capital infrastructure costs, the costs of clinical interpretation, or the health services associated with test ordering and/or follow-up care [53].
	Risk of unequal access to reimbursement by insurances	Bertier, et al. 2017 Johnson, et al. 2017 Casas 2018 Johnston, et al. 2018 Grebe, et al. 2020	Variations in insurance coverage, parental socioeconomic status, and geographic location are three factors that may limit access to germline genomic sequencing. The cost of testing and subsequent cancer screening may be overly burdensome for those with limited economic resources and poor insurance coverage. Efforts to integrate NGS into clinical practice should include advocacy for equitable access to genetic counseling, tumor, and germline sequencing and clinical follow-up as indicated based upon test results [54].
	Risk of biased treatment due to WGS result	Char 2015(a) Deem 2016 Bell, 2018 Graf, et al. 2019 Gyngell, et al. 2019	PGS- revealed findings may have unanticipated or unintended consequences for the individual patient, particularly the acutely ill patient: the potential to be used as justification to withhold certain therapeutic options; to decide the futility of others; to withdraw care; and, to ration scarce resources, such as organ transplantation, to one patient over another [55].
	Risk of reinforcing negative social attitudes towards disability	Deem 2016 Bell, 2018	However, several disability rights advocates have expressed concern that clinical use of genetic technologies may reinforce and perpetuate stigmatization of and discrimination against disabled persons in medical and social contexts. There is growing need, then, for clinicians and bioethicists to consider how the clinical use of WGS in the newborn period might exacerbate such harms to persons with disabilities [56].
	Risk of clinicians and bioethicists focus on common set of ethical issues (neglect other important ethical issues)	Deem 2016 Cabello, et al. 2021 Newson 2021	With respect to addressing specific ethical challenges that incidental findings pose to acquiring informed consent from patients or their families, the focus of clinicians and bioethicists tends to converge on a common set of issues. These include the patient’s or family’s preferences about which results will be returned, their understanding of the risks posed by routine data sharing and storage to their confidentiality and privacy, and their attitudes toward future use of genomic data and recontacting [56].
	Risk of researchers separating ethics from what they deem as purely scientific or technological actions	Abdul-Karim, et al. 2013 Thornock 2016	Researchers might be tempted to separate ethics from what they deem as purely scientific or technological actions. For instance, in WGS research, it may be tempting to see ethics as an integral component at the bookends of a study, at the beginning when obtaining consent or at the end when returning results, but see other steps (sequencing, analyzing, verification, storage) as wholly technological or scientific endeavors separate from ethics. However, these actions are not devoid of ethics because they are directly related to how researchers provide value to their stakeholders. Researchers should not assume that the storage of sequences is merely a technological or pragmatic necessity devoid of ethical obligations [57].
	Challenge to publish guidelines for standardized testing	Zawati, et al. 2014 Beale, et al. 2015 Bertier, et al. 2017 Rahimzadeh 2017	According to various authors, in order for WES to be offered in a standardized manner, formal guidelines, including strict quality control measurements, must be published. While some have called for this regulation to be provided by the Food and Drug Administration (in the USA), this may be challenging for regulators given the amount of data to be analyzed from a whole exome (about 30 million base pairs, or 1% of a whole genome) [28].
	Risk of outdated distinction between research and clinical care	Lunshof 2012 Botkin, et al. 2015 Newson and Schonstein 2016 Wouters, et al. 2017 Rotz and Kodish 2018 Byrjalsen, et al. 2020	Clinical application of research-stage procedures can save lives, as the exemplary case of the 15-month-old boy shows. In this case, the institutional review board-approved the use of nonvalidated experimental methods precisely because the primary purpose was to obtain a diagnosis for a patient; had the aim been gaining generalizable knowledge this would have turned it formally into research. This reasoning, however, is based on a questionable and probably outdated distinction between research and clinical care that takes systematic recording of outcomes as the decisive criterion for research. Moreover, can there be any instance of a diagnostic or therapeutic procedure – experimental or routine – that does not record results or yield generalizable knowledge? Also, clinical care and *n* = 1 studies are essentially connected. One could say that in ‘personalized’ medicine – and good medicine is always personalized – every medical intervention in an individual is a type of *n* = 1 study [58].
ISSUES RELATED TO PRETEST COUNSELLING			
Risk of unequal access to high-quality counselling		Sabatello and Appelbaum 2016 Bertier, et al. 2017 Rotz and Kodish 2018 Smith, et al. 2019 Szego, et al. 2019	For example, genomic counseling services may be more available in urban medical centers than in the rural setting. Genomic testing without genetic counseling is associated with a lack of informed decision making, misinterpretation of results and inappropriate clinical management, potential breaches of ethical standards, and adverse psychosocial outcomes [59].
Challenges for the informed consent process	Challenge of creating appropriate consent forms	Burke and Clarke 2016 Eno, et al. 2018 Hitchcock, et al. 2020	The challenges in creating appropriate consent forms are notable; there is a delicate balance in keeping the readability manageable while acknowledging a number of potential complications that may arise [20].
	Risk of traditional concept of informed consent no longer being feasible	Wilfond and Diekema 2012 Bunnik, et al. 2013 Bowdin, et al. 2016 Burke and Clarke 2016 Li, et al. 2016 Iskrov, et al. 2017 Newson 2017 Wouters, et al. 2017 Diamonstein 2019 Gore, et al. 2019 Gyngell, et al. 2019 Yu, et al. 2019 Byrjalsen, et al. 2020 Hitchcock, et al. 2020 Wolf, et al. 2020 Lynch, et al. 2021 Vears, et al. 2021	How can a valid, adequately informed consent be ensured given the volume and complexity of data generated, particularly with regard to incidental findings and variants of unknown significance? It may even be doubted that ‘informed consent’, as traditionally defined, is attainable in everyday practice [60].
	Different forms of required consent and amount of information depending on individual need	Burke and Clarke 2016 Li, et al. 2016 Diamonstein 2019	Informational requirements are complex and highly situated in terms of time, place and the individual situation in question. The informational needs of a family whose young child is undergoing investigation for severe impairment, as in case 1, who potentially will not attain medical decision-making autonomy, might differ from those such as the parents in case 2, particularly with regard to the management of incidental or uncertain information [60].
	Degree of directiveness (depending on clinical situation/urgency or personal need)	Botkin, et al. 2015 McCullough, et al. 2015 Wouters, et al. 2017 Diamonstein 2019 Gyngell, et al. 2019 Jamal, et al. 2020 Vears, et al. 2021	The nascent use of genomic testing in healthy individuals has also led some to argue that directive genetic counseling – where a professional takes a more active role in providing advice, guidance or recommendations – can be condoned. We suggest that directive genetic counseling may also be appropriate for at least some RGT in the NICU. While parents need to be able to both understand the possible outcomes of the test and should have the chance to reflect critically on their decision to have RGT, the known clinical utility of these tests means that the test can frequently have direct implications for subsequent treatment. This could be said to make RGT more like the kinds of medical tests that are routinely performed in NICU without explicit parental consent. However, given the possible implications for other family members, potential for future discrimination, combined with often uncertain direct benefit, gaining explicit consent to RGT remains prudent. Further, any directive counseling should not amount to coercion [44].
	Risk of undue influence regarding consent in research	Wilfond and Diekema 2012 Byrjalsen, et al. 2020	Finally, how should voluntariness and undue influence be understood in the context of assent? (e.g., does it matter if the research team offers the child $20 or if the parents offer to take the child for pizza if she agrees?) [61]
	Challenge of emotionally charged situation with high psychosocial needs	Oberg, et al. 2015 Li, et al. 2016 Rosell, et al. 2016 Clowes Candadai, et al. 2019 Diamonstein 2019 Gyngell, et al. 2019 Hill, et al. 2020 Lynch, et al. 2021	Participants described factors that contributed to their psychosocial needs, such as having a good HCP–parent relationship and HCPs’ consideration of parents’ well-being. […] Aspects such as trust and the provision of emotional support have been found to lead to more positive working relationships and less decisional conflict. Thus, it is important for HCPs to be mindful of the aspects of the relationship that have the potential to impact parents’ psychosocial needs and their decision making. Participants’ psychosocial needs may be as important as their informational need [62].
	Challenge for parents to really overlook decisions in advance	Abdul-Karim, et al. 2013 Burke and Clarke 2016 Wouters, et al. 2017 Vears, et al. 2020	To assert the ‘right not to know’ may be incoherent when it is not yet known that there is anything to (not) know, presenting a challenge to how advance instructions and preferences can be meaningfully established and respected [60].
	Risk that hope for cure makes parents consent to everything	Oberg, et al. 2015 Diamonstein 2019 Gore, et al. 2019 Gyngell, et al. 2019	With hopes for a cure, parental motivation to participate in WGS research may be high without fully understanding the range of results that may be returned, including variants of unknown significance and secondary findings [63].
	Risk of inflicted ought	Newson 2017 Malek, et al. 2019	The parents did not, however, hold a uniformly positive view of the choice to receive adult-onset SVs. Despite this, they felt a moral obligation to learn about SVs; that they would be ‘remiss … to not know what is knowable’. […] Anderson et al. reframe this as ‘inflicted ought’—some parents were given insight into genomic knowledge that they did not necessarily want to know, but felt they should nonetheless come to learn. […] It is also worth noting that the mere offer of a test may not be neutral. If a particular suite of information is being offered, that offer may be interpreted as implicit encouragement to accept it. This too could contribute to inflicted ought [26].
	Risk of too narrow understanding of autonomy	Newson 2017 Wouters, et al. 2017 Jamal, et al. 2020	There is a tendency in some bioethics discourse to construe autonomy superficially, such as presenting it as a property of decisions and inextricably tying it to informed consent. If a decision is supported by information and is made voluntarily with appropriate understanding, then it is said to be autonomous. However, this places too much emphasis on information [and its transfer] at the expense of the process of the decision and the psychological properties of the person involved [26].
	Challenge to determine how much choice parents should be given regarding which findings they can receive	Lunshof 2012 ACMG 2013 Bunnik, et al. 2013 May, et al. 2013 Bush 2014 Holm 2014 Holm, et al. 2014 Knoppers, Avard et al. 2014 Zawati, et al. 2014 Anderson, et al. 2015 Ayuso, et al. 2015 Beale, et al. 2015 Berg and Powell 2015 Botkin, et al. 2015 Clayton 2015 Hens and Dierickx 2015 Levenson 2015 McCullough, et al. 2015 Sénécal, et al. 2015 Botkin 2016 Bowdin, et al. 2016 Burke and Clarke 2016 Hufnagel, et al. 2016 Joseph, et al. 2016 Krabbenborg, et al. 2016 Kesserwan, et al. 2016 Newson and Schonstein 2016 Sabatello and Appelbaum 2016 Bertier, et al. 2017 Friedman, et al. 2017 Johnson, et al. 2017 Wouters, et al. 2017 Bell 2018 McGowan, et al. 2018 Vears, et al. 2018 Chassagne, et al. 2019 Cornelis and Wouters 2019 Hart, et al. 2019 Holm, et al. 2019 Ormond, et al. 2019 Ross and Clayton 2019 Wong, et al. 2019 Downie, et al. 2020 Hoell, et al. 2020 Savatt, et al. 2020 Sofer 2020 Vears 2021	The mainstream consensus of the bioethics community appears to be that adult-onset disorders with no effective prevention or treatment should be off-limits to parents and are most appropriate for informed decision-making by the individual when he or she becomes an adult. That being said, some argue that even these disorders fall within a parent’s responsibility to raise their child to the best of their ability and prepare them for any eventuality, that the theoretical harms are less significant than initially supposed and that parents are in the best position to make decisions relative to their child’s best interests [64].
	Challenge of parental decision making on behalf of child	Lunshof 2012 Wilfond and Diekema 2012 Abdul-Karim, et al. 2013 Holm 2014 Holm, et al. 2014 Knoppers, Avard, et al. 2014 Zawati, et al. 2014 Berg and Powell 2015 Botkin, et al. 2015 Clayton 2015 Hens and Dierickx 2015 Bowdin, et al. 2016 Newson and Schonstein 2016 Sabatello and Appelbaum 2016 Bertier, et al. 2017 Johnson, et al. 2017 Newson 2017 McGowan, et al. 2018 Rotz and Kodish 2018 Vears, et al. 2018 Cornelis and Wouters 2019 Gore, et al. 2019 Gyngell, et al. 2019 Chaudhari, et al. 2020 Hoell, et al. 2020 Dondorp, et al. 2021 Tibben, et al. 2021 Vears 2021	Indeed, application of principles regarding adult whole genome screening does not entail just implementing proxy consent and laying the burden of decision making with the caregivers. Children have special status in medical care: They are vulnerable in that they are dependent on others for their own health care. Respect for children as they are also includes respect for the fact that they will eventually grow up to be autonomous adults. Hence, choices made for them should in principle not rule out the possibility that they can make different choices in the future [65].
	Challenge to giving appropriate role to adolescents (capable of assent)	Wilfond and Diekema 2012 Abdul-Karim, et al. 2013 Holm, et al. 2014 Knoppers, Avard, et al. 2014 Zawati, et al. 2014 Ayuso, et al. 2015 Botkin, et al. 2015 Clayton 2015 Hens and Dierickx 2015 McCullough, et al. 2015 Sabatello and Appelbaum 2015 Bowdin, et al. 2016 Newson and Schonstein 2016 Sabatello and Appelbaum 2016 Johnson, et al. 2017 McGowan, et al. 2018 Cornelis and Wouters 2019 Gore, et al. 2019 Pervola, et al. 2019 Wong, et al. 2019 Hoell, et al. 2020 Lewis, et al. 2020 Dondorp, et al. 2021 Tibben, et al. 2021 Vears 2021	The common practice is for parents to determine what is in their children’s best interests, with adolescents at most asked to acquiesce. Even if we assume that most parents strive to make decisions that promote their children’s best interests, the lack of adolescents’ involvement raises the risk that parents’ views and anxieties—rather than those of the adolescent—will dominate the decision. Further complicating the situation is that as minors mature, they may hold values and preferences different from their parents’. How to balance parental authority against adolescents’ growing autonomy is not always clear [66].
	Risk of conflict of interests for parents	Berg and Powell 2015 Clayton 2015 Sabatello and Appelbaum 2015 Bowdin, et al. 2016 Sabatello and Appelbaum 2016 Bertier, et al. 2017 Holm, et al. 2019 Gyngell, et al. 2019 Tibben, et al. 2021	Finally, lack of adolescents’ involvement raises the risk that parents will conflate their interests and their adolescent’s interests, leading to SF-related decisions that reflect parents’ preferences (and anxieties) rather than those of the adolescent. […] Opinions are split, however, about returning SFs for carrier status with reproductive implications (e.g., carrier state for cystic fibrosis), disorders for which interventions will be deferred to adulthood (e.g., BRCA1/2), and adult-onset conditions without treatments that offer clear clinical benefit (e.g., Alzheimer disease). Whereas expert panels and professional guidelines generally suggest that these be deferred until adolescents reach maturity and can decide for themselves, studies indicate that many parents desire to learn all about their children’s genetic makeup. Although parents believe that it is their right and duty to access and manage their children’s genomic data, professionals often view themselves as the guardians of adolescents’ genomic-related rights in decisions that are intrinsically family-oriented. And whereas professionals call for distinctions based on medical utility and scientific validity, studies indicate that parents’ rationales may include not only personal and familial medical interests but also mere curiosity. Even if we assume that most parents (and professionals) strive to make decisions that promote children’s best interests, there is a risk that adolescents’ right (not) to know will not only be in conflict with familial interests, but also subjugated to parents’ (or others’) rights, interests and whims [41].
	Risk of conflict between parents	May, et al. 2013 Holm, et al. 2014 Berg and Powell 2015 Chassagne, et al. 2019 Holm, et al. 2019	In addition, even the decision making process could lead to strife between parents if they are unable to agree about whether or not to learn such information [64].
	Risk of conflict between HPCs/researchers and parents	McCullough, et al. 2015 Sabatello and Appelbaum 2016 Friedman, et al. 2019	Parents may have reasonable views about the implications of the best interests standard in its psychosocial dimensions for their child’s clinical care and well-being that differ from those of the child’s pediatrician for non–life-threatening conditions. In such cases, parents may appeal to values and beliefs that are not exclusively health-related when they more broadly conceptualize their child’s best interests. As a consequence, parents may reach an informed and considered judgment about the benefits and risks of receiving or not receiving results of genomic sequencing about non–life- threatening conditions that differ from the prima facie ethical obligations of pediatricians, as described above. Given the uncertainty of long-term psychological and social outcomes of genomic sequencing, parental judgments about psychosocial benefits and harms of such sequencing results typically will have as much authority as those of the pediatrician [67].
	Challenge due to complexity of issues	Wilfond and Diekema 2012 Bunnik, et al. 2013 Beale, et al. 2015 Berg and Powell 2015 Oberg, et al. 2015 Bowdin, et al. 2016 Burke and Clarke 2016 Deem 2016 Green, et al. 2016 Krabbenborg, et al. 2016 Lantos 2016 Li, et al. 2016 Bertier, et al. 2017 Werner-Lin, et al. 2018 Chassagne, et al. 2019 Clowes Candadai, et al. 2019 Cornelis and Wouters 2019 Diamonstein 2019 Gore, et al. 2019 Johnson, et al. 2019 Smith, et al. 2019 Yu, et al. 2019 Byrjalsen, et al. 2020 Hill, et al. 2020 Vears, et al. 2021	Genetic counselors (GCs) have expressed challenges with the length, complexity, and content of the GWS consent process. Specifically, GCs had difficulty ensuring their patients accurately understood the benefits, limitations, potential results, and implications of GWS for themselves and their family members […] Most participants thought that large volumes of information given at one time can result in “information overload” [62].
	Challenges due to time pressure/ time restraints	Bowdin, et al. 2016 Li, et al. 2016 Bertier, et al. 2017 Clowes Candadai, et al. 2019 Gyngell, et al. 2019 Sanderson, et al. 2019 Smith, et al. 2019 Hill, et al. 2020 Lynch, et al. 2021 Vears, et al. 2021	Initial reports described the informed consent process for pediatric WES as requiring 3–6 h. Recently, it has been suggested that the WES consent process could be shortened to 30–60 min depending on the type and timing of secondary analysis performed. In contrast, we find that the process of obtaining informed consent for WGS testing of children is complex and requires multiple encounters with genetics professionals, in part due to the predictive component of the test and the number of individuals potentially affected by test results [53].
	Challenge of expertise and training for effective communication	McCullough, et al. 2015 Burke and Clarke 2016 Li, et al. 2016 Sabatello and Appelbaum 2016 Bertier, et al. 2017 McGowan, et al. 2018 Clowes Candadai, et al. 2019 Diamonstein 2019 Gore, et al. 2019 Johnson, et al. 2019 Sanderson, et al. 2019 Smith, et al. 2019 Yu, et al. 2019	To ensure quality clinical practice, the ASHG recommends that HCPs involved with pediatric genetic testing need to have appropriate training. With the expected growth of genomic testing, there may be inadequate trained medical geneticists and counselors to support patients and families [62].
Challenges for what should be discussed/ Content	Explain to parents and families the potential for all the types of findings	ACMG 2013 Ayuso, et al. 2015 Blackburn, et al. 2015 Botkin, et al. 2015 Levenson 2015 McCullough, et al. 2015 Bowdin, et al. 2016 Burke and Clarke 2016 Li, et al. 2016 Rosell, et al. 2016 Bertier, et al. 2017 Johnson, et al. 2017 Wouters and Cornelis 2017 Bell 2018 Johnston, et al. 2018 Clowes Candadai, et al. 2019 Sanderson, et al. 2019 Yu, et al. 2019 Deignan, et al. 2020 Lalonde, et al. 2020 Vears, et al. 2020 Hay, et al. 2021	Clinicians must explain to parents and families the potential for all the types of findings [28].
	Potential for results to change over time	Burke and Clarke 2016 Deem 2016 Vears, et al. 2020	The potential impact that WGS will have on a newborn’s future medical management complicates the clinician’s task of ensuring that parental consent for testing is properly informed. Parental understanding of how diagnostic results might impact medical management is crucial to informed, responsible decisions about whether a child should receive WGS. Clinicians cannot rule out the possibility that incidental findings will have negative downstream effects on patients’ future medical care. Insofar as understanding the potential risks associated with WGS is a requirement for consent to be truly informed, clinicians who recommend WGS for ill newborns should counsel families not only about how the genomic information will be managed but also about the possibility that this information will have downstream effects on their child’s future options for clinical management. Part of appropriate clinical counseling, then, will involve informing parents that the clinical utility of uncovered variants may change over time, and that one implication of this change could be restriction of their child’s access to scarce medical resources [56].
	What does testing entail (needed samples, etc.)	Burke and Clarke 2016 Rosell, et al. 2016 Deignan, et al. 2020	Parents felt they needed to know: […] what did the testing entail (i.e. What types of samples were needed and what would be expected from them and their children) [68].
	Voluntary nature of the test	Burke and Clarke 2016 Malek, et al. 2019	Proposed minimal requirements for consent in whole genome sequencing: […] Voluntary nature of the test [60].
	Description of alternative diagnostic tests, if available	Burke and Clarke 2016	Proposed minimal requirements for consent in whole genome sequencing: […] Alternative test Description of alternative diagnostic tests, if available [69].
	Possibility of refusal at any time, without consequences for care	Burke and Clarke 2016	Proposed minimal requirements for consent in whole genome sequencing: […] Possibility of refusal at any time, without consequences for clinical or social care [69].
	Clarify parental values	Bowdin, et al. 2016 Li, et al. 2016 Wouters and Cornelis 2017 Bell 2018 Werner-Lin, et al. 2018 Gore, et al. 2019 Gyngell, et al. 2019 Malek, et al. 2019 Sanderson, et al. 2019 Smith, et al. 2019 Yu, et al. 2019 Tibben, et al. 2021 Vears 2021	The overall data highlighted the context-dependent nature of decision making for GWS. Participants’ diverse circumstances and other elements such as personality, values, beliefs, and amount of prior knowledge influenced and personalized their decision making and was believed by many participants to be a factor in the amount and type of information they needed. […] It is important for HCPs to assess parents’ individual values, priorities, and informational needs and tailor information accordingly [62].
	Privacy and confidentiality considerations	Burke and Clarke 2016 Bertier, et al. 2017 McGowan, et al. 2018 Lalonde, et al. 2020	Further, researchers ought to address privacy and confidentiality considerations in the informed consent process, being attentive to the risks of personally identifiable genomic research data making its way into medical, legal, or insurance environments [70].
	Potential implications of gathered knowledge for family members	Bowdin, et al. 2016 Burke and Clarke 2016 Krabbenborg, et al. 2016 Bertier, et al. 2017 Johnson, et al. 2017 Hart, et al. 2019 Yu, et al. 2019 Hill, et al. 2020 Hay, et al. 2021	Pretest counseling was another unsolved issue identified by technology users in the pediatric setting. Similarly, all three sets of guidelines address this issue, specifying a list of aspects that should be discussed when counseling patients and their families and conducting informed consent prior to WES. This generally includes a discussion of the expected outcomes of testing, outlining the potential benefits and risks of the test, the limitations of such testing, and the implications for family members [28].
	Potential effects on parent-child bonding	Gyngell, et al. 2019	Therefore, rather than focusing on whether information should be binned, tiered or something else, those obtaining consent to RGT should talk with parents to promote realistic expectations from testing. They should also engage them about the broad goal of the test, clarify parental values and hopes, canvass the possible impact of the test on bonding […] [44].
	Emotional preparedness to receive the potential results	Rosell, et al. 2016 Malek, et al. 2019 Yu, et al. 2019	Parents also felt it was important for the genetics team to explore with families whether they were ready or if they really wanted the information that may be obtained from the WES and to discuss with them the possibility that a positive result may still result in unanswered questions. Consenting for WES typically focuses on the technical facts (i.e., different types of variants) and likelihood of a diagnosis; findings from these parents suggest that the emotional aspect of the potential outcomes of a diagnosis should be explored as part of the WES consenting process [68].
	(Un) realistic expectations	Bowdin, et al. 2016 Burke and Clarke 2016 Rosell, et al. 2016 Bertier, et al. 2017 Johnson, et al. 2017 Wouters and Cornelis 2017 Johnston, et al. 2018 Werner-Lin, et al. 2018 Chassagne, et al. 2019 Gore, et al. 2019 Gyngell, et al. 2019 Lantos 2019 (a) Malek, et al. 2019 Smith, et al. 2019 Szego, et al. 2019Yu, et al. 2019 Hill, et al. 2020 Lalonde, et al. 2020 Vears, et al. 2020	Concerns have already been raised about the overly positive portrayal of WGS and WES, and the danger of this creating unrealistic expectations among the public. Therefore, rather than focusing on whether information should be binned, tiered or something else, those obtaining consent to RGT should talk with parents to promote realistic expectations from testing [44].
	Disclosing policy	Abdul-Karim, et al. 2013 Knoppers, Avard et al. 2014 McCullough, et al. 2015 Sénécal, et al. 2015 Burke and Clarke 2016 Bell 2018 Werner-Lin, et al. 2018 Deignan, et al. 2020	Clinician, researchers, and direct-to-consumer provider should describe to potential recipients incidental and secondary findings that are likely to arise or be sought from the tests and procedures conducted. Practitioners should inform potential recipients about their plan for disclosing and managing incidental and secondary findings, including what findings will and will not be returned [71].
	Reanalysis policy	Knoppers, Avard, et al. 2014 Johnson, et al. 2017	Mandatory reanalysis of genomic raw data is unlikely to occur on a broad scale due to resource limitations and loss of participants to follow-up; however, it may be pursued by individual centers or laboratories. Accordingly, any plan for reanalysis should be disclosed to patients during pretest counseling [54].
	Consent to data sharing for research	Ayuso, et al. 2015 Oberg, et al. 2015 Burke and Clarke 2016 Bertier, et al. 2017 Yu, et al. 2019	Proposed minimal requirements for consent in whole genome sequencing: […] Destination and potential further use of samples, such as research, retesting with further phenotypical information, retesting as genomic databases become more extensive [60].
	Duration of process until reception of results	Rosell, et al. 2016	Parents felt they needed to know: […] how long would testing take [68].
ISSUES RELATED TO SEQUENCING, ANALYSIS AND INTERPRETATION			
Challenges regarding infrastructure	Risk of inconsistencies and variability due to different bioinformatics pipelines	ACMG 2013 Ayuso, et al. 2015 McCullough, et al. 2015 Deem 2016 Green, et al. 2016 Bertier, et al. 2017 Johnson, et al. 2017 Eno, et al. 2018 Vears, et al. 2018 Horton and Lucassen 2019 Yu, et al. 2019	As one example, although an NGS-based test holds the potential to scan the entirety of the exome or genome, the laboratory may not be capable of analyzing all of the sequenced areas due to insufficient coverage of the base pairs in a targeted region. Unfortunately, accepted levels for depths of coverage remain to be determined as do thresholds for calling genetic variants (i.e. quality scores) during analysis of sequencing reads. When considering how specific germline variants are handled across laboratories, the metrics for interpreting, thresholds for classifying, and policies for reporting are not consistent. Although the ACMG has developed a plan for the classification and reporting of germline variants into one of five different categories (i.e. benign, likely benign, variants of uncertain significance, likely pathogenic, or pathogenic), there is an industry-wide lack of standardization in placing variants into one of these five categories. As a result, it is possible for a patient to receive reports with conflicting interpretations of variant pathogenicity from different clinical laboratories [54].
	Risk of lengthy turnaround times	Editorial 2013 Dimmock and Bick 2014 Krabbenborg, et al. 2016 Bertier, et al. 2017 Werner-Lin, et al. 2018 Smith, et al. 2019	Several parents expressed frustration with the length of time required to receive results, reporting that the length of time was the biggest drawback to their experience overall [72].
	Risk of lack of data analysts/ bioinformaticians	Beale, et al. 2015	Staffing numbers and training were also considered important by some interviewees, especially regarding the need for more bioinformaticians and genetic counsellors. Limited formal qualification-bearing education and training opportunities are being developed, but these take several years of highly specialised work to complete [46].
	Risk of lack of data sharing	ACMG 2013 Editorial 2013 Deem 2016 Bertier, et al. 2017 Vears, et al. 2018 Learned, et al. 2019	Technology users highlighted the need for systematic and generalized sharing of variant data from WES to enable the advancement of research and to enhance the detection of genetic causes of disease [28].
	Risk of lack of access to high quality databases	Deem 2016 Bertier, et al. 2017 Vears, et al. 2018 Learned, et al. 2019	Until there is a clinical-grade general database available to analysts and clinicians, as well as broadly accepted methods for standardizing interpretations of sequence variants, it will remain a challenge to ensure that clinicians are sufficiently familiar with and have ready access to the most recent emerging data about variant-disease associations and their clinical significance [56].
Challenge to determine how broad/targeted analysis		Bunnik, et al. 2013 Clayton, et al. 2014 Berg and Powell 2015 Botkin, et al. 2015 Botkin 2016 Thornock 2016 Bertier, et al. 2017 Johnson, et al. 2017 Vears, et al. 2018 Holm, et al. 2019 Ross and Clayton 2019 Deignan, et al. 2020	One way to avoid the need to deal with most incidental findings is by only examining genes that have a high likelihood of being associated with a child’s clinical condition. […] In order to increase the clinical and economic efficiency of sequencing, geneticists may analyze only those portions of the genome that have a high likelihood—based on prior studies—of being associated with the particular symptoms of the newborn. Though the entire genome is sequenced, the sequence is filtered so that only those variants related to a newborn’s symptoms are fully analyzed and returned to the treating physician. This approach only generates information about specific genes and as such it is less likely than a more comprehensive analysis of the genome to reveal unsought information. This approach will not avoid every incidental finding since there may be some incidental findings in the specific genes that are analyzed. But it will decrease the number of such findings. It will not, however, eliminate the need for difficult ethical choices [57].
Challenge to decide whether it should be actively searched for a certain list of disease-associated genes in every genome-wide sequencing		Bush 2014 Clayton, et al. 2014 Knoppers, Avard, et al. 2014 Anderson, et al. 2015 Botkin, et al. 2015 Clayton 2015 Levenson 2015 Sénécal, et al. 2015 Bowdin, et al. 2016 Burke and Clarke 2016 Johnson, et al. 2017 Wouters and Cornelis 2017 Vears, et al. 2018 Cornelis and Wouters 2019 Hart, et al. 2019 Holm, et al. 2019 Ormond, et al. 2019 Szego, et al. 2019 Ross and Clayton 2019 Wong, et al. 2019 De Wert, et al. 2021 Miller, et al. 2021 Wouldstra, et al. 2021	Based on the capability of NGS approaches to simultaneously sequence multiple genes at the same time, in 2013, the American College of Medical Genetics and Genomics (ACMG) recommended that clinical labor atories performing this testing analyze and report back on pathogenic variants for a panel of 56 disease-associated genes regardless of the original indication for ordering the testing. These genes were selected for analysis and reporting because it was felt that early identification of the associated diseases followed by intervention were likely to prevent serious morbidity and mortality. These recommendations were criticized for not allowing individuals to opt out of receiving these genetic results. Significant ethical concerns were also raised due to the potential to identify adult-onset conditions in children. There was also fear that an obligatory analysis of an additional 56 genes would significantly increase the time and resources required for testing. In response, the ACMG has revised this policy in 2014, relaxing their position around the mandatory nature of analysis and reporting on this prespecified list of genes. Recommendations for analysis and reporting are changing rapidly and in 2016, the ACMG again revised their position where the gene list now includes 59 genes. Currently, individual clinical laboratories determine the scope of tests that each offers, and many use a tiered informed consent that allows patients to choose which of these so-called ‘ACMG genes’ they would like to have tested [54].
Challenges for the interpretation of variants	Risk due to the gap between amount of data which are generated and knowledge to use them in a clinical context	Lunshof 2012 Editorial 2013 Knoppers, Sénécal, et al. 2014 [b] Landau, et al. 2014 Hens and Dierickx 2015 Howard, et al. 2015 McCullough, et al. 2015 Petrikin, et al. 2015 Reinstein 2015 Bowdin, et al. 2016 Deem 2016 Lantos 2016 Newson and Schonstein 2016 Rosell, et al. 2016 Friedman, et al. 2017 Iskrov, et al. 2017 Johnson, et al. 2017 Johnston, et al. 2018 Horton and Lucassen 2019 Lantos 2019(a) Lantos 2019(b) Szego, et al. 2019	In both sequencing approaches (WGS and WES), however, differentiating disease-related mutations from variations of unknown clinical significance is a major problem, even in the known coding regions of genes. In WGS this represents an even greater challenge in the non-coding parts of the genome where function is not yet clearly defined for many sequences. Thus, the clinical significance of thousands of genomic and exomic variants detected by NGS cannot presently be interpreted with complete certainty, preventing evidence-based decisions being made to guide treatment and clinical surveillance [73].
	Risk to miss scientific developments	Deem 2016 Lantos 2016 Newson and Schonstein 2016 Lantos 2019(a) Lantos 2019(b) Odgis, et al. 2021	It is difficult to maintain up-to-date information on every known genetic disease. No centrally maintained repository of all rare and disease-associated variants currently exist [74].
	Risks due to limited ethnic diversity within the sequencing content of reference databases	Green, et al. 2016 Johnson, et al. 2017 Rotz and Kodish 2018 Chaudhari, et al. 2020 Odgis, et al. 2021	Racial and ethnic differences may play a role in the heterogeneity of cancer biology. Differences in research participation may lead to future disparities in our fundamental understanding of malignancy and our ability to offer precision treatment approaches. For example, as part of the National Institutes of Health–sponsored Cancer Genome Atlas project, white patients were overrepresented, and Asian and Hispanic patients were underrepresented, compared with the entire United States population. Due to decreased research participation with less background data, individuals from minority groups are at a higher risk for false-positive and false-negative genomic testing results. Increasing the availability of genomic tests to historically underserved populations is critical to the amelioration of health disparities and ensuring distributive justice [59].
	Risks of uncertainties and failures in the classification of variants	Tarini and Goldenberg 2012 ACMG 2013 Editorial 2013 Holm, et al. 2014 Landau, et al. 2014 Petrikin, et al. 2015 Reinstein 2015 Sénécal, et al. 2015 Bowdin, et al. 2016 Deem 2016 Kesserwan, et al. 2016 Newson and Schonstein 2016 Friedman, et al. 2017 Johnson, et al. 2017 Newson 2017 Johnston, et al. 2018 Rotz and Kodish 2018 Vears, et al. 2018 Lantos 2019(b) Gyngell, et al. 2019 Horton and Lucassen 2019	Our understanding of the clinical significance of any given sequence variant falls along a gradient, ranging from those in which the variant is almost certainly pathogenic for a disorder to those that are almost certainly benign.” To classify genetic variants along this spectrum, interpreters must take into consideration population data on the frequency of the allele in question, computational and predictive data (“in silico models”) in which a deleterious effect from the variant is suggested, functional studies, family history, and studies revealing that people with the variant have the disease and those without the variant do not. It is a laborious process that can be considered as much an art as a science. The art requires value judgments about the risks of “calling” a variant as pathogenic. There are risks to both false-positives and false-negatives [49].
	Risk of information overload	Lunshof 2012 Botkin and Rothwell 2016 Joseph, et al. 2016 Lantos 2016 Sabatello and Appelbaum 2016 Johnston, et al. 2018 Lantos 2019(a)	Biesecker recognized the problems of information overload, saying, “A whole-genome or whole- exome result is overwhelming for both the clinician and the patient … [because] variants from [the] genome or exome range from those that are extremely likely to cause disease to those that are nearly certain to be benign and every gradation between these 2 extremes.” [74]
ISSUES RELATING TO COMMUNICATING RESULTS			
Challenges for reporting results from lab to clinician/participant/patients/parents	Risks due to inconsistencies in guidelines and IRB decisions which results to report	Sénécal, et al. 2015 Eno, et al. 2018 Vears, et al. 2018 Vears 2021	Although NGS technologies are well-embedded in the clinical setting for identification of genetic causes of disease, guidelines issued by professional bodies are inconsistent regarding some aspects of reporting results. Most recommendations do not give detailed guidance about whether variants of uncertain significance (VUS) should be reported by laboratory personnel to clinicians, and give conflicting messages regarding whether unsolicited findings (UF) should be reported [75]. The management and disclosure of incidental pediatric genomic research findings are increasingly pressing issues. Investigators are looking to IRBs and research ethics consultants for guidance, yet disagreements persist about this complicated set of issues. As NGS becomes increasingly widespread, excessive IRB variation may lead to decisional inconsistencies. More uniform guidelines on how to address the disclosure of incidental findings can serve as a framework to guide IRB decisions, and additional investigation is needed [16].
	Risk of ineffective communication between lab and ordering physician	Abdul-Karim, et al. 2013 Eno, et al. 2018 Vears, et al. 2018	In order for a targeted approach to be effective, using either targeted gene-capture or exome sequencing with bioinformatic filtering, the laboratory must have a clear idea of the clinical question that is being asked. Therefore, detailed and accurate information about the clinical phenotype of the patient is required to determine which genes should be included in the analysis. Detailed phenotypic information is also crucial for deciding which variants to report. Although laboratories can contact the referring clinician after sequencing takes place to get more details about the patient’s phenotype, and how this relates to the variants identified, this additional step can be time consuming and would be unnecessary if more information was provided at the time of the request. We suggest it is not good clinical practice for laboratories to perform exome or large panel sequencing until sufficient clinical information is received from the clinician [75].
	Challenge to decide whether results should be made directly available to patients/ participants (incl. Raw data)	ACMG 2013 Editorial 2013 Johnson, et al. 2017 Eno, et al. 2018 Grebe, et al. 2020 Beauvais, et al. 2021	Laboratory tests are ordered by clinicians based on the medical needs of their patients, and the results are typically returned to the referring clinician. Only recently have laboratories been either encouraged or required to make results directly available to patients. Patients who seek out their laboratory test results independent of their health-care providers have made their own choice about learning these results [14].
	Challenge to determine where to stop knowledge in different steps of information pipeline	Bunnik, et al. 2013 Hufnagel, et al. 2016 Friedman, et al. 2017 Eno, et al. 2018 Vears, et al. 2018 Holm, et al. 2019 Deignan, et al. 2020	Highlighted in this case is also the ethical dilemma that occurs when laboratory personnel are privy to information that would be relevant to an individual but are unable to act on it. Some feel that if laboratory personnel have knowledge of clinically important information about a patient or participant or even a family member whose biological specimen was submitted for validation testing, they ought to disclose it or at least discuss it with the ordering physician or researcher [21].
Challenges for Post-test-counselling	Risks due to varying degrees of genetic literacy among clinicians	Howard, et al. 2014 Deem 2016 Johnson, et al. 2017 Vears, et al. 2018 Werner-Lin, et al. 2018 Chaudhari, et al. 2020 Deuitch, et al. 2020 Vears, et al. 2020	The working group discussed whether part of the role of the referring clinician is to filter the variants that are reported to them and to decide which of those variants it is appropriate to report to the patient. This is likely to be particularly challenging when the referring specialist does not have specific training in genetics. It is unrealistic to expect laboratories to tailor their reports to the experience of the referring clinician [52].
	Risk of overestimating genetic results	Dimmock and Bick 2014 Howard, et al. 2014 Reinstein 2015 Burke and Clarke 2016 Johnston, et al. 2018 Gyngell, et al. 2019	An ethical consideration is what life is best worth living. Do we support genetic determinism in which all information is present at the beginning with a clear path and destiny and no ultimate freedom of choice? [73]
	Challenge to deal with “genotype only” situations	McCullough, et al. 2015 Johnston, et al. 2018 Horton and Lucassen 2019 Lantos 2019(b) Szego, et al. 2019	With our current imperfect state of knowledge, it may be hard to know whether a finding of a pathogenic variant in an asymptomatic patient ought to be considered a false-positive test result or whether, instead, such a test result should be considered a warning flag indicating a higher-than-average probability that the person will develop disease in the future. Some have referred to people in this situation as “patients- in-waiting.” I would like to suggest, in a manner only partially tongue-in- cheek, that such a situation could also be characterized as a “false-negative phenotype.” With either label, the implications are that the patient will be treated as if they were at risk for developing disease in the future. This may lead to increased anxiety, extra diagnostic testing, and, in some cases, even treatment of disease that may or may not ever occur […] Such testing leads to questions about how to think about the concept of “molecular diagnoses” in apparently healthy people. Given a genomic variant that would be classified as likely pathologic in an apparently healthy person, there are 2 possibilities. The tests could be wrong. Or the people could, in fact, have disease but have not yet developed phenotypic manifestations of illness [49].
	Risk of therapeutic uncertainty despite diagnosis	Tarini and Goldenberg 2012 McCullough, et al. 2015 Chassagne, et al. 2019 Szego, et al. 2019	The downside is that in some instances, technology creates a “therapeutic gap” by making it possible to screen for a disorder before effective treatments are available. The imminence of financially feasible whole-genome sequencing (WGS) is likely to transform this gap into a chasm [76].
	Challenge to decide to whom report: parents or children/adolescents or together?	Sénécal, et al. 2015 Sabatello and Appelbaum 2016 Sundby, et al. 2018 Werner-Lin, et al. 2018	We recommend providers disclose findings to adolescents and parents together, and in each stage offer the opportunity for parents and adolescents to meet separately to discuss concerns, questions, and next steps. Separating family members to discuss these issues will minimize protective buffering, enhance informed consent, and support long term care of the identified patient. A significant feature distinguishing pediatric from adult care is the role of parents in directing the care of their children and providing consent on behalf of the child. Beliefs, emotions, or concerns revealed in separate conversations may also alert providers to the presence of red flags for depression or anxiety, and enable a referral to family counseling. In some families, it might be appropriate to first return results to the parents, and then discuss results with the parents and child together, and then with the child separately [72].
	Risk of negative impact of diagnosis on parent-child bonding	ACMG 2013 Holm 2014 Allain 2015 Berg and Powell 2015 Bowdin, et al. 2016 Burke and Clarke 2016 Johnson, et al. 2017 Johnston, et al. 2018 Gyngell, et al. 2019 Hill, et al. 2020	One consideration that parents may not appreciate when they agree to testing is the potential for WGS and WES in the newborn period to interfere with family dynamics by influencing parent-child bonding. These concerns could be exacerbated in the cases of RGT in the NICU, given the short turnaround time. While parent-child bonding starts during pregnancy, it intensifies in the months after birth. This means that RGT in the NICU will often return genomic results very early in the bonding process, whereas traditional (slower) testing in unwell infants will return results when bonding is established [44].
	Challenge to identify when a prognosis is sufficiently poor that treatment may be withheld	Gyngell, et al. 2019 Horton and Lucassen 2019	RGT also raises significant ethical challenges. Some of these are shared with other prognostic tests and technologies, and some are shared with perennial questions around the care of very unwell newborns.19 These include normative uncertainty, and the challenge of identifying when a prognosis is sufficiently poor that treatment may be withheld, or sufficiently good that it must not be [44].
	Risks regarding variants of unknown significance	ACMG 2013 Ayuso, et al. 2015 Oberg, et al. 2015 Deem 2016 Joseph, et al. 2016 Lantos 2016 Rosell, et al. 2016 Bertier, et al. 2017 Friedman, et al. 2017 Johnson, et al. 2017 Vears, et al. 2018 Werner-Lin, et al. 2018 Gore, et al. 2019 Gyngell, et al. 2019 Scollon, et al. 2019 Szego, et al. 2019 Vears, et al. 2020 Hay, et al. 2021	Because a VUS cannot be confirmed as benign and may therefore be related to the patient’s condition, they can generate anxiety in patients. The assessment of their pathogenicity may require testing of other family members, or additional investigations that may be costly and time consuming for patients and their families […] Indeed, the status of such VUS is likely to change as research in genomics advances. Jiang and colleagues suggested that this reevaluation should be offered to patients as part of comprehensive care and patient follow- up. However, Biesecker and Green explained that the potential for a “negative result” to become “positive” or clinically relevant could complicate the post-counseling process [28].
	Risk to undermine right not to know with obligatory disclosure of UFs	ACMG 2013 Borry, et al. 2014 Holm, et al. 2014 Knoppers, Avard, et al. 2014 Zawati, et al. 2014 Anderson, et al. 2015 Blackburn, et al. 2015 Botkin, et al. 2015 Levenson, 2015 Botkin 2016 Bowdin, et al. 2016 Burke and Clarke 2016 Bertier, et al. 2017 Ormond, et al. 2019 Ross and Clayton 2019 Benedetti and Marron 2021 Vears 2021	Parents should be able to decline secondary findings in advance of testing, but clinicians should disclose those findings if they indicate a serious health risk and “effective action can be taken to mitigate that threat,” the statement recommends. “We said it’s OK not to look for secondary findings, but if a lab does find something with serious implications, the clinician must disclose it,” says Jeffrey Botkin, MD, MPH, first author of the ASHG position statement. […] ASHG clearly states that a clinician has a fiduciary duty to override parent preferences to not receive secondary results when genomic sequencing reveals a serious risk to children’s health and medical action can mitigate the threat, Dr. Biesecker notes. “I think this is correct and a significant advance in the thinking on this topic,” he adds [15].
	Risk to undermine child’s future autonomy with disclosure of UFS of adult onset conditions and carrier status	Lunshof, 2012 ACMG 2013 Borry, et al. 2014 Holm 2014 Holm, et al. 2014 Allain 2015 Anderson, et al. 2015 Blackburn, et al. 2015 Knoppers, Avard, et al. 2014 Levenson 2015 McCullough, et al. 2015 Sénécal, et al. 2015 Bowdin, et al. 2016 Burke and Clarke 2016 Kesserwan, et al. 2016 Lantos 2016 Sabatello and Appelbaum 2016 Bertier, et al. 2017 Johnson, et al. 2017 Johnston, et al. 2018 Garrett, et al. 2019 Lantos 2019(a) Ormond, et al. 2019 Ross and Clayton 2019 Wong, et al. 2019 Savatt, et al. 2020 Sofer 2020 De Wert, et al. 2021 Dondorp, et al. 2021 Miller, et al. 2021 Tibben, et al. 2021 Uveges and Holm 2021	Whereas guidelines from the British Medical Association‍ and the American Academy of Pediatrics‍ recommended that carrier status results obtained incidentally should be conveyed to parents, the American Medical Association‍ and the German Society of Human Genetics recommended that this information should not be disclosed to parents or other third parties. Miller et al.‍. write, “The provision of carrier or predictive genetic testing is seen to infringe on the child’s autonomy and right to confidentiality because it forecloses on the child’s right to decide whether to seek this information and to whom it should be disclosed.” [74]
	Challenge to balance the best interests of the child with the best interests of the family regarding the disclosure of UFs	Abdul-Karim, et al. 2013 ACMG 2013 Borry, et al. 2014 Bush 2014 Clayton, et al. 2014 Holm 2014 Holm, et al. 2014 Knoppers, Avard, et al. 2014 Zawati, et al. 2014 Anderson, et al. 2015 Berg and Powell 2015 Clayton 2015 McCullough, et al. 2015 Sénécal, et al. 2015 Botkin 2016 Botkin and Rothwell 2016 Bowdin, et al. 2016 Burke and Clarke 2016 Kesserwan, et al. 2016 Newson and Schonstein 2016 Bertier, et al. 2017 Friedman, et al. 2017 Johnson, et al. 2017 Johnston, et al. 2018 Vears, et al. 2018 Cornelis and Wouters 2019 Garrett, et al. 2019 Holm, et al. 2019 Horton and Lucassen 2019 Ross and Clayton 2019 Szego, et al. 2019 Wong, et al. 2019 Chaudhari, et al. 2020 Savatt, et al. 2020 Sofer 2020 De Wert, et al. 2021 Miller, et al. 2021 Uveges and Holm 2021	A counter argument to ‘respect for an open future’ considers that genomic testing of the child in the present may be the only way to identify familial genetic risks; an argument that considers present day interests of the family over the child’s future interests and autonomy. If the child does not survive to adulthood to make an autonomous decision to undergo testing, then he or she does not benefit from being allowed an open future. The family, however, is potentially harmed through the failure to identify an inherited genetic predisposition that would only be identified through testing of the child. Given the possibility to reveal information about genetic risk in family members, whose best interests should prevail? Should the child’s immediate medical well-being [i.e. his or her self-regarding or present day interests] be weighed over the benefits that the family would receive through the child’s test results? […] Children exist within a family unit; therefore, some argue that best interest evaluations should occur within the context of overall family interests [54].
	Challenge to define “actionable” UFs	Abdul-Karim, et al. 2013 Holm, et al. 2014 Hens and Dierickx 2015 Green, et al. 2016 Kesserwan, et al. 2016 Ormond, et al. 2019	Especially in the case of minors, questions regarding what is actionable and what is not remain. For example, the finding of an extra X chromosome in males (Klinefelter syndrome) may on the one hand be significant in that it is related to an increased chance of learning problems or autism. On the other hand, the only constant in Klinefelter syndrome is infertility. Is this enough to warrant inclusion of sex chromosome screening in the panel? Knowledge of infertility may avoid medical odysseys later in life, but may also be part of the genetic privacy of the child that needs safeguarding. What about mutations or copy number variants that are linked with an increased risk of autism? [65]
	Challenge to deal with UF of misattributed parentage	Holm, et al. 2014 Botkin, et al. 2015 Johnson, et al. 2017 Bell 2018 Eno, et al. 2019 Deignan, et al. 2020	Another potential harm is the possibility of disrupting family relationships with unanticipated genetic information. One example involves the identification of nonpaternity (i.e. the person claiming to be the father of the child is not the biological father). It can be difficult to decide whether and how to release this result to the patient and family and currently, there is not a consensus within the ethics literature as how best to proceed [54].
	Challenge to deal with UF of consanguinity	Botkin, et 1 l. 2015	The ASHG recommends that laboratories adopt data standards and analytical methods that allow reliable detection of incest. Practitioners should develop procedures for case management when genetic laboratory results are consistent with incest involving a minor. Practitioners have a duty to report suspected child abuse. Health-care providers do not have a responsibility to report incest involving consenting adults, even though this might be illegal in their jurisdiction [45].
	Challenge for parents to understand and deal with the results	Bowdin, et al. 2016 Rosell, et al. 2016 Lantos 2016 Rogers and Zhang 2016 Bertier, et al. 2017 Johnson, et al. 2017 Werner-Lin, et al. 2018 Lantos 2019(a) Malek, et al. 2019Scollon, et al. 2019 Vears, et al. 2020	CGES findings may present difficulties for families and patients due to poor genome- based health literacy, lack of tolerance for ambiguity or uncertainty, or the emotional demand and future implications of testing in the context of a child’s ongoing and intensive clinical care [72].
	Need for good relationship between parents and clinicians	Rosell, et al. 2016 Werner-Lin, et al. 2018 Scollon, et al. 2019	We also found that post-test genetic counseling and clinical follow-up are critically important. The clinical relationship, based on trust, respect, and open communication was key to how many parents perceived the process of WES and parents both with and without reportable findings wanted closer contact with the clinicians [68].
	Need for thoughtful communication of results	Goldenberg and Sharp 2012 Abdul-Karim, et al. 2013 Holm, et al. 2014 Blackburn, et al. 2015 Krabbenborg, et al. 2016 McCullough, et al. 2015 Sénécal, et al. 2015 Bowdin, et al. 2016 Deem 2016 Rosell, et al. 2016 Sabatello and Appelbaum 2016 Werner-Lin, et al. 2018 Chassagne, et al. 2019 Gyngell, et al. 2019 Lantos 2019(a) Malek, et al. 2019 Scollon, et al. 2019 Smith, et al. 2019 Odgis, et al. 2021 Sachdev, et al. 2021	There should be designated supports for communication of information that may be disappointing or concerning for participants while respecting participant preferences to receive such information [77].
Risk of legal liability for disclosure/non-disclosure of findings		Green, et al. 2016 Bertier, et al. 2017	CSER investigators have also conducted important legal and regulatory analyses relevant to clinical sequencing, including the legal liability for disclosure or non-disclosure of findings to patients, research participants, and family members [78].
Risk of lacking post-test strategies and resources (access to genetic counselling, clinical follow-up, treatment options required etc.)		Jenkins, et al. 2008 Abdul-Karim, et al. 2013 Holm, et al. 2014 Ayuso, et al. 2015 Sénécal, et al. 2015 Bowdin, et al. 2016 Joseph, et al. 2016 Kesserwan, et al. 2016 Krabbenborg, et al. 2016 Rosell, et al. 2016 Bertier, et al. 2017 Johnson, et al. 2017 Bell 2018 Werner-Lin, et al. 2018 Chassagne, et al. 2019 Holm, et al. 2019 Deuitch, et al. 2020 Hitchcock, et al. 2020 Cabello, et al. 2021	There must be a process of immediate follow-up and querying of participants, combined with an offer of genetic counselor involvement for further questions or concerns [77].
ISSUES RELATED TO FUTURE USE OF DATA			
Challenges of data sharing, storage and governance	Challenge to determine data ownership	Lunshof 2012 Editorial 2013 Botkin, et al. 2015 Clayton 2015 Sabatello and Appelbaum 2016	Other issues relate to genomic data-sharing by professionals, especially in the context of new informational technologies. One such issue is the increasing incorporation of genomic data in electronic medical records. This development has been intensely debated, given that these records may optimize personalized care, but their “multi-owner and multi-user nature” may increase the risks of privacy breaches and misuses of genomic data. […] Studies regarding pediatric genomic SFs show that although parents often worry that their children’s participation in research will lead to loss of privacy [and possible stigma and discrimination], they do not view their own access to their children’s genomic information as a privacy concern. Many parents in fact disclose genetic data about their children to extended family members, friends, neighbors, and others, suggesting a sense of ownership [41].
	Risk to privacy	Lunshof 2012 Wilfond and Diekema 2012 Editorial 2013 Knoppers, Avard, et al. 2014 Clayton 2015 Howard, et al. 2015 Oberg, et al. 2015 Sabatello and Appelbaum 2015 Bowdin, et al. 2016 Joseph, et al. 2016 Rogers and Zhang 2016 Sabatello and Appelbaum 2016 Bertier, et al. 2017 Friedman, et al. 2017 Seidel 2017 Johnston, et al. 2018 Vears, et al. 2018 Zacharias, et al. 2018 Lantos 2019(a) Lewis, et al. 2020 Beauvais, et al. 2021 Benedetti and Marron 2021	However, data sharing can be ethically challenging, particularly with regards to issues of privacy and confidentiality. Although data may be anonymised or deidentified, the nature of genomic information means that it is potentially reidentifiable, particularly in patients with rare diseases. In these cases, linking phenotypic data with genomic data is crucial for determining whether a variant is causative. However, this makes these patients more easily identifiable [75].
	Risk of discrimination	Dimmock and Bick 2014 Knoppers, Sénécal, et al. 2014 Allain 2015 Oberg, et al. 2015 Bowdin, et al. 2016 Deem 2016 Joseph, et al. 2016 Rogers and Zhang 2016 Sabatello and Appelbaum 2016 Bertier, et al. 2017 Friedman, et al. 2017 Johnson, et al. 2017 Johnston, et al. 2018 Zacharias, et al. 2018 Graf, et al. 2019 Szego, et al. 2019 Chaudhari, et al. 2020 Grebe, et al. 2020 Savatt, et al. 2020 Sofer 2020 Benedetti and Marron 2021 Miller, et al. 2021 Sachdev, et al. 2021	Other important challenges to returning secondary WES/WGS findings are the imperfect confidentiality of genetic information and uneven regulation of the use of personal genetic data by employers, insurers, corporations and governments. While many countries and jurisdictions legally constrain discrimination on the basis of genetic pre-disposition to disease (e.g. the Genetic Information Nondiscrimination Act (GINA) in the United States), other countries lack specific laws [e.g. Canada] and existing legal protections may fall short of covering every situation in which confidentiality is breeched or genetic information used to deny employment and/or insurance [53].
	Risk irresponsible parental data sharing	Allain 2015 Clayton 2015 Sabatello and Appelbaum 2015 Sabatello and Appelbaum 2016 Beauvais, et al. 2021	There is evidence that the concept of genetic privacy is applied asymmetrically within families. Whereas many parents disclose genetic data about their children to extended family members, friends, neighbors, and others, studies of adults who have undergone predictive testing show that they are wary of disclosing their own genetic results [66].
	Challenge to determine whose responsibility it is to initiate reanalysis, provide access and recontact patients/parents/participants	Abdul-Karim, et al. 2013 Knoppers, Avard, et al. 2014 Ayuso, et al. 2015 Botkin, et al. 2015 Levenson 2015 McCullough, et al. 2015 Sénécal, et al. 2015 Bowdin, et al. 2016 Burke and Clarke 2016 Kesserwan, et al. 2016 Thornock, 2016 Bertier, et al. 2017 Johnson, et al. 2017 Vears, et al. 2018 Cornelis and Wouters 2019 Chaudhari, et al. 2020	It has been argued that clinicians have a responibility to recontact patients when new information, regarding the interpretation of genetic information, becomes available. No consensus currently exists about how this should be managed, and addressing such concerns is likely to be particularly challenging in the paediatric setting: how should information be communicated to children gaining competency and autonomy with regard to their own health? Any duty to recontact children in adulthood, to ensure the appropriate communication of results or to reconsider the significance of variants of unknown significance in the light of new information, would be challenging from logistical, economic and legal perspectives [60].
	Challenge to gain re-consent of children when they reach majority	Wilfond and Diekema 2012 Knoppers, Avard, et al. 2014 Howard, et al. 2015 Lantos 2016 Bertier, et al. 2017 Lantos 2019(a) Wong, et al. 2019	Finally, the issue of when (if ever) to seek reconsent from people who were enrolled as minors but have now reached the age of majority is only beginning to be addressed. Some argue that parents should never be able to consent for the enrollment of minors.‍ Others suggest that a robust process of recontact and reconsent at the age of majority will be sufficient.‍ Given the rapid pace of change in the field, it is difficult to anticipate what we may be doing 5, 10, or 18 years from now both in terms of genomics and in terms of our ability to stay in touch with research subjects [74].
	Risk of storage costs being higher than sequencing again in the future	Knoppers, Sénécal, et al. 2014 Howard, et al. 2015 Friedman, et al. 2017	Others argue that the cost of secure storage and stewardship of these data over the lifetime of the child may exceed the cost of repeating the genomic testing in the future if the information becomes necessary [79].
Challenge to determine which type of data should be included in the medical record		Botkin, et al. 2015 Oberg, et al. 2015 Chaudhari, et al. 2020 Deignan, et al. 2020 Grebe, et al. 2020	Recent federal regulations provide for laboratory results to be the property of the patient, raising questions about how much genomic information should be placed in the medical record, particularly in the case of genetic variation that does not have well-established clinical implications [45].

## Discussion

To our knowledge, this is the first systematic qualitative review of the full spectrum of ethical issues in pediatric genome-wide sequencing discussed in the literature. Most ethical issues identified in relation to genome-wide sequencing typically reflect ethical issues that arise in general genetic testing in children [80–82], but they are often amplified by the increased quantity of data obtained, and associated uncertainties.

The challenges surrounding UFs are a good example of this and were one of the most frequently discussed ethical issues in the literature. UFs have intensified tremendously in genome-wide sequencing, as the likelihood to generate them and their sheer number have increased a lot. These challenges are even bigger and more in pediatric genomic-sequencing as parents then have to make the decisions regarding the reception of UFs for their child. Issues frequently identified in this review include how much choice parents should be given regarding which findings they want to receive for their children; the risk to undermine the right not to know with an obligatory disclosure of UFs; the challenge of interpreting and balancing the best interests of the child with the best interests of the family regarding the disclosure of UFs (see Table 2). The two ethical principles, which are important to consider when debating the challenges regarding the return of UFs, are beneficence and autonomy. The principle of respect for autonomy includes the right that everybody generally should decide intentionally, with understanding and without substantial external influence. Beneficence implies the idea of ‘doing well’ and acting in someone’s best interests [27]. In pediatric genome-wide sequencing, the application of these principles is complicated as decisions are mostly made on behalf of the children. Parents are usually granted the authority to make decisions believed to be in the best interests of their child. However, children have a developing capacity to make autonomous decisions for themselves, and most will have full capacity for autonomous decision-making in the future as adults. Hence, in decision-making regarding UFs in pediatric genomic sequencing, important ethical concepts that recur, might compete and are weighed against each other are the right not to know, the child’s right to an open future and the best interests principle. Potential conflicts can occur between the parental opinion of what is in the best interests of the child, the healthcare professional’s view of what is in the best interests of the child, the parental authority to make decisions for their children, the child’s future autonomy, and the parent’s view of what is in their own best interests. The right not to know can be endangered, for example, if children/adolescents are not involved in the decision-making process and are suddenly confronted with knowledge about an UF; or also if parents unintentionally learn about their own health status through their children’s findings. The child’s right to an open future captures the idea that a pediatric patient in the future will have the capacity to exercise his/her autonomy and that this right should be preserved for them: when parents make decisions for their children now, they should do it in a way that allows the child the greatest possibility to make a decision for her−/himself in the future as adults [83]. It is particularly cited when it comes to UFs of conditions that do not present in childhood. This is especially challenging when these UFs could at the same time be directly relevant for the parents: a frequently cited example is the detection of a BRCA gene mutation, which does not pose an immediate health risk for children themselves, because it only becomes relevant in adulthood; but which could possibly result in immediate medical measures for the parents and avert danger for them [21, 54, 84]. In these cases, the best interests principle is often brought up and discussed whether the consideration of the child’s best interests includes his/her interests to have healthy parents.

The question can be posed whether we should treat medical information from the genome fundamentally different than we treat other medical information (an idea that is often summarized under the term genetic exceptionalism [85–87]) and whether the “right not to know” has special weight and role in the context of pediatric genome-wide sequencing. Several authors in the last years have discussed and criticized an absolute right not to know and argued for a more nuanced application of it, also in genomics [88–91].

In the discussion following the descriptive presentation of the ethical issues identified in the literature, such as ours, it should not be forgotten that these ethical issues have different qualities, risks and practical relevance and therefore require different solutions: First, there are ethical challenges, such as discrimination or unequal access to reimbursement, which could be solved by legal or policy measures (as, for example, the Genetic Information Nondiscrimination Act of the USA aims to do [92]). Second, there are issues raised that should be examined empirically, such as questions of clinical utility and cost-effectiveness or concerns about parental distress. For example, there have been several empirical studies examining the issues of parental distress recently [93] suggesting that these issues may not be of significant concern to those affected. Finally, there are also genuine ethical dilemmas, such as around the right not to know vs. the right to know in the context of UFs, which can only be approached by weighing up the context-specific risks and benefits. It is important to keep in mind that the ethical risks involved and the ethical issues identified are not of all the same importance in every situation, do not carry the same ethical risk and do not all have the same practical implications.

With the increasing use of genome-wide sequencing, non-genetic medical specialties, such as pediatricians, are also increasingly confronted with it: They are often the first to see and know the affected patient and their families best; they provide pre- and post-test care; they are able to make a referral to a geneticist or, in some countries, can order genome-wide sequencing of their pediatric patients themselves. However, studies show that given the complexity of genome-wide sequencing, pediatricians are often uncomfortable with it [94]. Here, not only further training opportunities are required, but also intense and fruitful interdisciplinary cooperation between the various professionals involved is of great importance in order to ensure high-quality long-term care for patients and their families – and to avoid overburdening the various medical specialists involved.

With the increased complexity and potentially difficult ethical decisions associated with genome-wide sequencing, the difficulty of responsibly designing and conducting the informed consent process also increases enormously. It has even been argued that the traditional idea of informed consent might no longer be feasible [38, 60, 61] given the multitude of possible outcomes and complexities and that, at least in certain clinical situations, more directive patient/parent counselling could be necessary [24, 44, 95]. In any case, this means that the importance of good communication and genetic counselling has increased dramatically to enable patients/parents to make decision as well-informed as possible. Providing the necessary resources here, in terms of finance, personnel and time, is of great importance. These points are also supported by the results of the literature analysis provided by Bertier et al. [28] analyzing ‘unsolved challenges in pediatric whole-exome sequencing’ discussed by technology users. Their analysis also emphasizes that counselling presents a major challenge for health care professionals due to the high complexity of issues and that training for effective communication was needed to best enable the patient and his/her family to make informed decisions. Furthermore, they also stress the particular challenges in pediatric genome-wide sequencing as parents here make the decisions on their children’s behalf and due to the higher likelihood to obtain UFs.

Despite the long list of ethical issues, awareness of some appears to be higher than others, as these are discussed a lot more often, questions and challenges around UFs being the most prominent. This observation is supported by the systematic review of technology users view’s about clinical WES by Bertier et al. [30], which reports UFs to be among the three most raised challenges and a steadily increasing proportion of articles debating these. The fact that the only two other systematic reviews that were among our search results [2, 29] were exclusively dedicated to the topic of UFs also indicates that there is a clear focus of the ethical debate on this topic. On the one hand, this is understandable because, as described above, the topic of UFs poses major challenges, particularly in the pediatric context. On the other hand, this concentration of the debate on a few ethical issues might harbor the danger that ethical challenges in the context of genome-wide sequencing are too quickly equated with the topic of unsolicited findings, and thus other equally important points are neglected. For example, one aspect that is only discussed in three texts of our review is collected in the subcode *Risk of irresponsible parental data sharing* [41, 42, 66]. For a comprehensive debate and responsible use of genome-wide sequencing it is important though, that users are aware of the full spectrum of ethical challenges. This systematic review is also intended to contribute to this end. Of course, this does not mean that all aspects are always equally relevant for every case, but it always depends on the specific situation (e.g. newborns vs. almost adults; seriously ill in emergency situations vs. a disorder where there is medical emergency, etc.). Furthermore, although awareness of the full spectrum of ethical issues is important, they should be balanced against the enormous potential benefits of pediatric genome-wide sequencing in a context-specific manner.

What makes the discussion of the ethical aspects even more difficult, especially for other specialists/non-geneticists, is the confusing terminology. With regard to both the test procedure/ the scope of analysis, as well as with the topic of UFs [24, 96, 97], there is a multitude of terms, some of which are used synonymously, while other authors clearly distinguish them from each other. Thus, for example, for this review it was decided to use the term genome-wide sequencing instead of the term whole genome/exome sequencing used in most articles, since the technology does not even cover the *whole* exome or genome and furthermore, in most cases of clinical application only a part of the sequenced data is actually looked at. In addition, the term ‘whole’ runs the risk of obscuring the fact that a large part of the data collected cannot yet be meaningfully clinically interpreted and of raising unrealistic expectations not only for patient’s parents but also for non-geneticist medical physicians.

In light of the increased complexity of genome-wide sequencing, including of the ethical challenges, it is of great importance that the necessary resources, financially, in terms of personnel and also in terms of time, are available. This is the only way to ensure that genome-wide sequencing is used responsibly, that despite the described complexity the decisions of patients and parents are made as informed as possible, and that they are also well cared for in the long term. This also includes good cooperation between all professionals involved and sufficient further training opportunities, e.g. for pediatricians, as they are becoming increasingly involved in testing and will play a key role in providing information, support and follow-up for patients and their families. The comprehensive overview of ethical issues, provided in this review, can inform educational material and raise awareness among practitioners and serve as a check-list helping parents and their pediatricians to obtain more information.

### Limitations

One limitation of this review might be seen in the fact that the searches were restricted to PubMed and Google Books with relevancy ranking. It is true that although the review is systematic, not all the existing literature dealing with ethical issues concerning genome-wide sequencing might have been included. However, this is not to be considered an overly disadvantageous factor and the approach was considered to be appropriate for various reasons: the search strategy allowed thematic saturation, and the publications that were finally analysed covered journals from all relevant fields (medicine, public health, nursing, social science and philosophy); additionally, former systematic reviews [98, 99] in the bioethics field, which based their research on additional databanks such as EMBASE, CINAHL or Euroethics, found few additional references. Furthermore, as the articles relevant to this review’s pediatric focus were extracted at a later step of our literature search, hence our search algorithm was not specific to the pediatric context. However, it is believed that this made our search more comprehensive. One could further note that our spectrum will not comprehensively give guidance on how to deal with the issues addressed. There are two main reasons for the restriction to the descriptive presentation of the ethical issues. First, the aim is to provide an evidence base for the further assessment of ethical issues. Hence, neither the relevance of single ethical issue is evaluated, nor the best solutions for each issue determined. Second, there are currently no best practice standards for the development of practice recommendation for ethical issues [33]. This includes the lack of well-established methods for the critical appraisal of ethical issues themselves or the corresponding sources/literature. Additionally, because of the heterogeneous use of terms for genome-wide sequencing technologies, it might be possible that some publications were not identified.

## Conclusion

This review gives a comprehensive overview of ethical issues in pediatric genome-wide sequencing which are discussed in the literature. It can inform educational material and raise awareness among practitioners. Ethical issues related to the analysis of human DNA in the context of clinical care and research have been discussed continually for the past 50 years. Most issues are not new as such but multiplied and amplified by genome-wide sequencing. This review is a first step to map the huge variety of issues. This is particularly important as awareness of the possibilities, but also the challenges, of genome-wide sequencing for children is becoming increasingly urgent also for other medical fields, i.e. non-geneticists. It highlights the importance that the medical genetics and ethics communities together with other medical professions involved work jointly on specific case related guidelines to grant the maximum benefit for the care of the children, while preventing patient harm and disproportionate overload of clinicians and the healthcare system by the wealth of available options and economic incentives to increase testing.

## Supplementary Information

**Additional file 1.** Bibliographical Information of Analysed Publications. A list providing full bibliographical information of all 87 publications included in the analysis of this systematic review.

## Data Availability

The datasets used and/or analysed during the current study available from the corresponding author on reasonable request.

## Abbreviations

HCP: Health care professional
UF: Unsolicited Findings
WES: Whole exome sequencing
WGS: Whole genome sequencing
