# Supplementary material for: The full spectrum of ethical issues in pediatric genome-wide sequencing: a systematic qualitative review
Source: BMC Pediatr. 2021 Sep 6;21:387. doi: 10.1186/s12887-021-02830-w (PMC8420043; doi:10.1186/s12887-021-02830-w)
Supplement: Supplementary file 1 — Additional file 1. Bibliographical Information of Analysed Publications. A list providing full bibliographical information of all 87 publications included in the analysis of this systematic review. [file 12887_2021_2830_MOESM1_ESM.docx]

# **Additional Table 1** Bibliographical Information of Analysed Publications

| **No.** | **Publication** |
| --- | --- |
| 1 | Abdul-Karim R, Berkman BE, Wendler D, Rid A, Khan J, Badgett T, et al. Disclosure of incidental findings from next-generation sequencing in pediatric genomic research. Pediatrics. 2013;131(3):564-71. |
| 2 | Allain DC. Testing Children for Adult-Onset Disorders. In: Berliner JL, editor. Ethical Dilemmas in Genetics and Genetic Counseling Principles through Case Scenarios: Oxford University Press; 2015. p. 96-116. |
| 3 | American College of Medical Genetics and Genomics (ACMG). Incidental findings in clinical genomics: a clarification. Genetics in medicine: official journal of the American College of Medical Genetics. 2013;15(8):664-6. |
| 4 | Anderson JA, Hayeems RZ, Shuman C, Szego MJ, Monfared N, Bowdin S, et al. Predictive genetic testing for adult-onset disorders in minors: a critical analysis of the arguments for and against the 2013 ACMG guidelines. Clin Genet. 2015;87(4):301-10. |
| 5 | Ayuso C, Millan JM, Dal-Re R. Management and return of incidental genomic findings in clinical trials. Pharmacogenomics J. 2015;15(1):1-5. |
| 6 | Beale S, Sanderson D, Sanniti A, Dundar Y, Boland A. A scoping study to explore the cost-effectiveness of next-generation sequencing compared with traditional genetic testing for the diagnosis of learning disabilities in children. Health Technol Assess. 2015;19(46):1-90. |
| 7 | Beauvais MJS, Thorogood AM, Szego MJ, Sénécal K, Zawati MH, Knoppers BM. Parental Access to Children's Raw Genomic Data in Canada: Legal Rights and Professional Responsibility. Frontiers in genetics. 2021;12:535340. |
| 8 | Bell SG. Ethical Implications of Rapid Whole-Genome Sequencing in Neonates. Neonatal Netw. 2018;37(1):42-4. |
| 9 | Benedetti DJ, Marron JM. Ethical Challenges in Pediatric Oncology Care and Clinical Trials. Recent results in cancer research Fortschritte der Krebsforschung Progres dans les recherches sur le cancer. 2021;218:149-73. |
| 10 | Berg JS, Powell CM. Potential Uses and Inherent Challenges of Using Genome-Scale Sequencing to Augment Current Newborn Screening. Cold Spring Harb Perspect Med. 2015;5(12). |
| 11 | Bertier G, Sénécal K, Borry P, Vears DF. Unsolved challenges in pediatric whole-exome sequencing: A literature analysis. Critical reviews in clinical laboratory sciences. 2017;54(2):134-42. |
| 12 | Blackburn HL, Schroeder B, Turner C, Shriver CD, Ellsworth DL, Ellsworth RE. Management of Incidental Findings in the Era of Next-generation Sequencing. Curr Genomics. 2015;16(3):159-74. |
| 13 | Boardman FK, Hale R, Young PJ. Newborn screening for haemophilia: The views of families and adults living with haemophilia in the UK. Haemophilia: the official journal of the World Federation of Hemophilia. 2019;25(2):276-82. |
| 14 | Borry P, Shabani M, Howard HC. Is There a Right Time to Know? The Right Not to Know and Genetic Testing in Children. J Law Med Ethics. 2014;42(1):19-27. |
| 15 | Botkin JR. Ethical issues in pediatric genetic testing and screening. Curr Opin Pediatr. 2016;28(6):700-4. |
| 16 | Botkin JR, Belmont JW, Berg JS, Berkman BE, Bombard Y, Holm IA, et al. Points to Consider: Ethical, Legal, and Psychosocial Implications of Genetic Testing in Children and Adolescents. American journal of human genetics. 2015;97(1):6-21. |
| 17 | Botkin JR, Rothwell E. Whole Genome Sequencing and Newborn Screening. Curr Genet Med Rep. 2016;4(1):1-6. |
| 18 | Bowdin SC, Hayeems RZ, Monfared N, Cohn RD, Meyn MS. The SickKids Genome Clinic: developing and evaluating a pediatric model for individualized genomic medicine. Clin Genet. 2016;89(1):10-9. |
| 19 | Bunnik EM, de Jong A, Nijsingh N, de Wert GM. The new genetics and informed consent: differentiating choice to preserve autonomy. Bioethics. 2013;27(6):348-55. |
| 20 | Burke K, Clarke A. The challenge of consent in clinical genome-wide testing. Arch Dis Child. 2016;101(11):1048-52. |
| 21 | Burke LW. Disclosure of Genome Sequencing Results: Are Pediatricians Ready? Pediatrics. 2015;136(4):e1005-6. |
| 22 | Bush L. In the best interest of the child: psychological and ethical reflections on traditions, contexts, and perspectives in pediatric clinical genomics. The American journal of bioethics : AJOB. 2014;14(3):16-8. |
| 23 | Byrjalsen A, Stoltze UK, Castor A, Wahlberg A. Germline whole genome sequencing in pediatric oncology in Denmark-Practitioner perspectives. Molecular genetics & genomic medicine. 2020;8(8):e1276. |
| 24 | Cabello JF, Novoa F, Huff HV, Colombo M. Expanded Newborn Screening and Genomic Sequencing in Latin America and the Resulting Social Justice and Ethical Considerations. International journal of neonatal screening. 2021;7(1). |
| 25 | Casas KA. Adoptees' Pursuit of Genomic Testing to Fill Gaps in Family Health History and Reduce Healthcare Disparity. Narrat Inq Bioeth. 2018;8(2):131-5. |
| 26 | Char D(a). Preventive Genomic Sequencing and Care of the Individual Patient. The American journal of bioethics : AJOB. 2015;15(7):32-3. |
| 27 | Char D(b). Whole-genome sequencing in critically ill infants and emerging ethical challenges. Lancet Respir Med. 2015;3(5):333-5. |
| 28 | Chassagne A, Pélissier A, Houdayer F, Cretin E, Gautier E, Salvi D, et al. Exome sequencing in clinical settings: preferences and experiences of parents of children with rare diseases (SEQUAPRE study). European journal of human genetics: EJHG. 2019;27(5):701-10. |
| 29 | Chaudhari BP, Manickam K, McBride KL. A pediatric perspective on genomics and prevention in the twenty-first century. Pediatric research. 2020;87(2):338-44. |
| 30 | Clayton EW. How Much Control Do Children and Adolescents Have over Genomic Testing, Parental Access to Their Results, and Parental Communication of Those Results to Others? J Law Med Ethics. 2015;43(3):538-44. |
| 31 | Clayton EW, McCullough LB, Biesecker LG, Joffe S, Ross LF, Wolf SM. Addressing the ethical challenges in genetic testing and sequencing of children. The American journal of bioethics : AJOB. 2014;14(3):3-9. |
| 32 | Clowes Candadai SV, Sikes MC, Thies JM, Freed AS, Bennett JT. Rapid clinical exome sequencing in a pediatric ICU: Genetic counselor impacts and challenges. Journal of genetic counseling. 2019;28(2):283-91. |
| 33 | Cornelis C, Wouters RHP. Genome Sequencing in Pediatrics: Ethical Issues. In: Biesecker BB, Tibben A, editors. Clinical Genome Sequencing Psychological considerations: Academic Press; 2019. p. 143-56. |
| 34 | Deem MJ. Whole-Genome Sequencing and Disability in the NICU: Exploring Practical and Ethical Challenges. Pediatrics. 2016;137 Suppl 1:S47-55. |
| 35 | Deignan JL, Chao E, Gannon JL, Greely HT, Hagman KDF, Mao R, et al. Points to consider when assessing relationships (or suspecting misattributed relationships) during family-based clinical genomic testing: a statement of the American College of Medical Genetics and Genomics (ACMG). Genetics in medicine: official journal of the American College of Medical Genetics. 2020;22(8):1285-7. |
| 36 | Deuitch N, Soo-Jin Lee S, Char D. Translating genomic testing results for pediatric critical care: Opportunities for genetic counselors. Journal of genetic counseling. 2020;29(1):78-87. |
| 37 | De Wert G, Dondorp W, Clarke A, Dequeker EMC, Cordier C, Deans Z, et al. Opportunistic genomic screening. Recommendations of the European Society of Human Genetics. European journal of human genetics: EJHG. 2021;29(3):365-77. |
| 38 | Diamonstein CJ. Factors complicating the informed consent process for whole exome sequencing in neonatal and pediatic intensive care units. Journal of genetic counseling. 2019;28(2):256-62. |
| 39 | Dimmock DP, Bick DP. Ethical issues in DNA sequencing in the neonate. Clin Perinatol. 2014;41(4):993-1000. |
| 40 | Dondorp W, Bolt I, Tibben A, De Wert G, Van Summeren M. 'We Should View Him as an Individual': The Role of the Child's Future Autonomy in Shared Decision-Making About Unsolicited Findings in Pediatric Exome Sequencing. Health care analysis: HCA: journal of health philosophy and policy. 2021. |
| 41 | Downie L, Halliday J, Lewis S, Lunke S, Lynch E, Martyn M, et al. Exome sequencing in newborns with congenital deafness as a model for genomic newborn screening: the Baby Beyond Hearing project. Genetics in medicine: official journal of the American College of Medical Genetics. 2020;22(5):937-44. |
| 42 | Editorial. Sequenced from the start. Nature. 2013;501(7466):135. |
| 43 | Eno C, Bayrak-Toydemir P, Bean L, Braxton A, Chao EC, El-Khechen D, et al. Misattributed parentage as an unanticipated finding during exome/genome sequencing: current clinical laboratory practices and an opportunity for standardization. Genetics in medicine : official journal of the American College of Medical Genetics. 2019;21(4):861-6. |
| 44 | Friedman JM, Bombard Y, Cornel MC, Fernandez CV, Junker AK, Plon SE, et al. Genome-wide sequencing in acutely ill infants: genomic medicine's critical application? Genetics in medicine : official journal of the American College of Medical Genetics. 2019;21(2):498-504. |
| 45 | Friedman JM, Cornel MC, Goldenberg AJ, Lister KJ, Sénécal K, Vears DF. Genomic newborn screening: public health policy considerations and recommendations. BMC Med Genomics. 2017;10(1):9. |
| 46 | Garrett JR, Lantos JD, Biesecker LG, Childerhose JE, Chung WK, Holm IA, et al. Rethinking the "open future" argument against predictive genetic testing of children. Genetics in medicine: official journal of the American College of Medical Genetics. 2019;21(10):2190-8. |
| 47 | Genetti CA, Schwartz TS, Robinson JO, VanNoy GE, Petersen D, Pereira S, et al. Parental interest in genomic sequencing of newborns: enrollment experience from the BabySeq Project. Genetics in medicine: official journal of the American College of Medical Genetics. 2019;21(3):622-30. |
| 48 | Goldenberg AJ, Sharp RR. The ethical hazards and programmatic challenges of genomic newborn screening. Jama. 2012;307(5):461-2. |
| 49 | Gore RH, Bridges JFP, Cohen JS, Biesecker BB. Challenges to informed consent for exome sequencing: A best-worst scaling experiment. Journal of genetic counseling. 2019;28(6):1189-97. |
| 50 | Graf M, Char D, Hanson-Kahn A, Magnus D. Use of genetic risks in pediatric organ transplantation listing decisions: A national survey. Pediatric transplantation. 2019;23(4):e13402. |
| 51 | Grebe TA, Khushf G, Chen M, Bailey D, Brenman LM, Williams MS, et al. The interface of genomic information with the electronic health record: a points to consider statement of the American College of Medical Genetics and Genomics (ACMG). Genetics in medicine: official journal of the American College of Medical Genetics. 2020;22(9):1431-6. |
| 52 | Green RC, Goddard KAB, Jarvik GP, Amendola LM, Appelbaum PS, Berg JS, et al. Clinical Sequencing Exploratory Research Consortium: Accelerating Evidence-Based Practice of Genomic Medicine. American journal of human genetics. 2016;98(6):1051-66. |
| 53 | Gyngell C, Newson AJ, Wilkinson D, Stark Z, Savulescu J. Rapid Challenges: Ethics and Genomic Neonatal Intensive Care. Pediatrics. 2019;143(Suppl 1):S14-s21. |
| 54 | Hart MR, Biesecker BB, Blout CL, Christensen KD, Amendola LM, Bergstrom KL, et al. Secondary findings from clinical genomic sequencing: prevalence, patient perspectives, family history assessment, and health-care costs from a multisite study. Genetics in medicine: official journal of the American College of Medical Genetics. 2019;21(5):1100-10. |
| 55 | Hay E, Cullup T, Barnicoat A. A practical approach to the genomics of kidney disorders. Pediatric nephrology (Berlin, Germany). 2021. |
| 56 | Hens K, Dierickx K. Double Trouble: Preventive Genomic Sequencing and the Case of Minors. The American journal of bioethics : AJOB. 2015;15(7):30-1. |
| 57 | Hill M, Hammond J, Lewis C, Mellis R, Clement E, Chitty LS. Delivering genome sequencing for rapid genetic diagnosis in critically ill children: parent and professional views, experiences and challenges. European journal of human genetics : EJHG. 2020;28(11):1529-40. |
| 58 | Hitchcock EC, Study C, Elliott AM. Shortened consent forms for genome-wide sequencing: Parent and provider perspectives. Molecular genetics & genomic medicine. 2020;8(7):e1254. |
| 59 | Hoell C, Wynn J, Rasmussen LV, Marsolo K, Aufox SA, Chung WK, et al. Participant choices for return of genomic results in the eMERGE Network. Genetics in medicine: official journal of the American College of Medical Genetics. 2020;22(11):1821-9. |
| 60 | Holm IA. Clinical Management of Pediatric Genomic Testing. Curr Genet Med Rep. 2014;2(4):212-5. |
| 61 | Holm IA, McGuire A, Pereira S, Rehm H, Green RC, Beggs AH. Returning a Genomic Result for an Adult-Onset Condition to the Parents of a Newborn: Insights From the BabySeq Project. Pediatrics. 2019;143(Suppl 1):S37-s43. |
| 62 | Holm IA, Savage SK, Green RC, Juengst E, McGuire A, Kornetsky S, et al. Guidelines for return of research results from pediatric genomic studies: deliberations of the Boston Children's Hospital Gene Partnership Informed Cohort Oversight Board. Genetics in medicine : official journal of the American College of Medical Genetics. 2014;16(7):547-52. |
| 63 | Horton RH, Lucassen AM. Recent developments in genetic/genomic medicine. Clinical science (London, England: 1979). 2019;133(5):697-708. |
| 64 | Howard HC, Knoppers BM, Cornel MC, Wright Clayton E, Sénécal K, Borry P. Whole-genome sequencing in newborn screening? A statement on the continued importance of targeted approaches in newborn screening programmes. Eur J Hum Genet. 2015;23(12):1593-600. |
| 65 | Hufnagel SB, Martin LJ, Cassedy A, Hopkin RJ, Antommaria AH. Adolescents' preferences regarding disclosure of incidental findings in genomic sequencing that are not medically actionable in childhood. American journal of medical genetics Part A. 2016;170(8):2083-8. |
| 66 | Iskrov G, Ivanov S, Wrenn S, Stefanov R. Whole-Genome Sequencing in Newborn Screening-Attitudes and Opinions of Bulgarian Pediatricians and Geneticists. Front Public Health. 2017;5:308. |
| 67 | Jamal L, Schupmann W, Berkman BE. An ethical framework for genetic counseling in the genomic era. Journal of genetic counseling. 2020;29(5):718-27. |
| 68 | Jenkins MM, Rasmussen SA, Moore CA, Honein MA. Ethical issues raised by incorporation of genetics into the National Birth Defects Prevention Study. Am J Med Genet C Semin Med Genet. 2008;148c(1):40-6. |
| 69 | Johnson LM, Hamilton KV, Valdez JM, Knapp E, Baker JN, Nichols KE. Ethical considerations surrounding germline next-generation sequencing of children with cancer. Expert Rev Mol Diagn. 2017;17(5):523-34. |
| 70 | Johnson LM, Sykes AD, Lu Z, Valdez JM, Gattuso J, Gerhardt E, et al. Speaking genomics to parents offered germline testing for cancer predisposition: Use of a 2-visit consent model. Cancer. 2019;125(14):2455-64. |
| 71 | Johnston J, Lantos JD, Goldenberg A, Chen F, Parens E, Koenig BA. Sequencing Newborns: A Call for Nuanced Use of Genomic Technologies. The Hastings Center report. 2018;48 Suppl 2(Suppl 2):S2-s6. |
| 72 | Joseph G, Chen F, Harris-Wai J, Puck JM, Young C, Koenig BA. Parental Views on Expanded Newborn Screening Using Whole-Genome Sequencing. Pediatrics. 2016;137 Suppl 1:S36-46. |
| 73 | Kesserwan C, Friedman Ross L, Bradbury AR, Nichols KE. The Advantages and Challenges of Testing Children for Heritable Predisposition to Cancer. Am Soc Clin Oncol Educ Book. 2016;35:251-69. |
| 74 | King JS, Smith ME. Whole-Genome Screening of Newborns? The Constitutional Boundaries of State Newborn Screening Programs. Pediatrics. 2016;137 Suppl 1:S8-15. |
| 75 | Knoppers BM, Avard D, Sénécal K, Zawati MH. Return of whole-genome sequencing results in paediatric research: a statement of the P3G international paediatrics platform. Eur J Hum Genet. 2014;22(1):3-5. |
| 76 | Knoppers BM, Sénécal K, Borry P, Avard D. Whole-genome sequencing in newborn screening programs. Sci Transl Med. 2014;6(229):229cm2. |
| 77 | Krabbenborg L, Schieving J, Kleefstra T, Vissers LE, Willemsen MA, Veltman JA, et al. Evaluating a counselling strategy for diagnostic WES in paediatric neurology: an exploration of parents' information and communication needs. Clin Genet. 2016;89(2):244-50. |
| 78 | Lalonde E, Rentas S, Lin F, Dulik MC, Skraban CM, Spinner NB. Genomic Diagnosis for Pediatric Disorders: Revolution and Evolution. Frontiers in pediatrics. 2020;8:373. |
| 79 | Landau YE, Lichter-Konecki U, Levy HL. Genomics in newborn screening. J Pediatr. 2014;164(1):14-9. |
| 80 | Lantos JD. Ethical and psychosocial issues in whole-genome sequencing for newborns In: Demkow U, Płoski R, editors. Clinical Applications for Next-Generation Sequencing: Academic Press; 2016. p. 295-300. |
| 81 | Lantos JD(a). Ethical and Psychosocial Issues in Whole Genome Sequencing (WGS) for Newborns. Pediatrics. 2019;143(Suppl 1):S1-s5. |
| 82 | Lantos JD(b). The False-negative Phenotype. Pediatrics. 2019;143(Suppl 1):S33-s6. |
| 83 | Learned K, Durbin A, Currie R, Kephart ET, Beale HC, Sanders LM, et al. Barriers to accessing public cancer genomic data. Scientific data. 2019;6(1):98. |
| 84 | Levenson, D. American society of human genetics updates guidance on genetic testing in children: Group addresses predictive genetic testing, use of secondary findings from genomic sequencing tests. American journal of medical genetics Part A. 2015;167a(10):viii-ix. |
| 85 | Levy HL. Ethical and Psychosocial Implications of Genomic Newborn Screening. International journal of neonatal screening. 2021;7(1). |
| 86 | Lewis C, Hammond J, Hill M, Searle B, Hunter A, Patch C, et al. Young people's understanding, attitudes and involvement in decision-making about genome sequencing for rare diseases: A qualitative study with participants in the UK 100, 000 Genomes Project. European journal of medical genetics. 2020;63(11):104043. |
| 87 | Li KC, Birch PH, Garrett BM, MacPhee M, Adam S, Friedman JM. Parents' Perspectives on Supporting Their Decision Making in Genome-Wide Sequencing. J Nurs Scholarsh. 2016;48(3):265-75. |
| 88 | Lunshof JE. Whole genomes, small children, big questions. Per Med. 2012;9(7):667-9. |
| 89 | Lynch F, Nisselle A, Gaff CL, McClaren B. Rapid acute care genomics: Challenges and opportunities for genetic counselors. Journal of genetic counseling. 2021;30(1):30-41. |
| 90 | Malek J, Pereira S, Robinson JO, Gutierrez AM, Slashinski MJ, Parsons DW, et al. Responsibility, culpability, and parental views on genomic testing for seriously ill children. Genetics in medicine: official journal of the American College of Medical Genetics. 2019;21(12):2791-7 |
| 91 | May T, Zusevics KL, Strong KA. On the ethics of clinical whole genome sequencing of children. Pediatrics. 2013;132(2):207-9. |
| 92 | McCullough LB, Brothers KB, Chung WK, Joffe S, Koenig BA, Wilfond B, et al. Professionally Responsible Disclosure of Genomic Sequencing Results in Pediatric Practice. Pediatrics. 2015;136(4):e974-82. |
| 93 | McGowan ML, Prows CA, DeJonckheere M, Brinkman WB, Vaughn L, Myers MF. Adolescent and Parental Attitudes About Return of Genomic Research Results: Focus Group Findings Regarding Decisional Preferences. J Empir Res Hum Res Ethics. 2018;13(4):371-82. |
| 94 | Miller DT, Lee K, Gordon AS, Amendola LM, Adelman K, Bale SJ, et al. Recommendations for reporting of secondary findings in clinical exome and genome sequencing, 2021 update: a policy statement of the American College of Medical Genetics and Genomics (ACMG). Genetics in medicine: official journal of the American College of Medical Genetics. 2021. |
| 95 | Moultrie RR, Paquin R, Rini C, Roche MI, Berg JS, Powell CM, et al. Parental Views on Newborn Next Generation Sequencing: Implications for Decision Support. Maternal and child health journal. 2020;24(7):856-64. |
| 96 | Newson AJ. Whole genome sequencing in children: ethics, choice and deliberation. Journal of medical ethics. 2017;43(8):540-2. |
| 97 | Newson AJ. The promise of public health ethics for precision medicine: the case of newborn preventive genomic sequencing. Human genetics. 2021. |
| 98 | Newson AJ, Schonstein L. Genomic Testing in The Paediatric Population: Ethical Considerations in Light of Recent Policy Statements. Mol Diagn Ther. 2016;20(5):407-14. |
| 99 | Oberg JA, Glade Bender JL, Cohn EG, Morris M, Ruiz J, Chung WK, et al. Overcoming challenges to meaningful informed consent for whole genome sequencing in pediatric cancer research. Pediatr Blood Cancer. 2015;62(8):1374-80. |
| 100 | Odgis JA, Gallagher KM, Suckiel SA, Donohue KE, Ramos MA, Kelly NR, et al. The NYCKidSeq project: study protocol for a randomized controlled trial incorporating genomics into the clinical care of diverse New York City children. Trials. 2021;22(1):56. |
| 101 | Ormond KE, O'Daniel JM, Kalia SS. Secondary findings: How did we get here, and where are we going? Journal of genetic counseling. 2019;28(2):326-33. |
| 102 | Pervola J, Myers MF, McGowan ML, Prows CA. Giving adolescents a voice: the types of genetic information adolescents choose to learn and why. Genetics in medicine: official journal of the American College of Medical Genetics. 2019;21(4):965-71. |
| 103 | Petrikin JE, Willig LK, Smith LD, Kingsmore SF. Rapid whole genome sequencing and precision neonatology. Semin Perinatol. 2015;39(8):623-31. |
| 104 | Rahimzadeh V. Sharing Outside the Sandbox? The Child’s Right to an Open Data Sharing Future in Genomics and Personalized Medicine. In: Verma M, Barh D, editors. Progress and Challenges in Precision Medicine: Academic Press; 2017. p. 171-85. |
| 105 | Reinstein E. Challenges of using next generation sequencing in newborn screening. Genet Res (Camb). 2015;97:e21. |
| 106 | Robinson JO, Wynn J, Biesecker B, Biesecker LG, Bernhardt B, Brothers KB, et al. Psychological outcomes related to exome and genome sequencing result disclosure: a meta-analysis of seven Clinical Sequencing Exploratory Research (CSER) Consortium studies. Genetics in medicine: official journal of the American College of Medical Genetics. 2019;21(12):2781-90. |
| 107 | Rogers Y-H, Zhang C. Genomic Technologies in Medicine and Health: Past, Present, and Future. In: Kumar D, Antonarakis S, editors. Medical and Health Genomics: Academic Press; 2016. p. 15-28. |
| 108 | Rosell AM, Pena LD, Schoch K, Spillmann R, Sullivan J, Hooper SR, et al. Not the End of the Odyssey: Parental Perceptions of Whole Exome Sequencing (WES) in Pediatric Undiagnosed Disorders. J Genet Couns. 2016;25(5):1019-31. |
| 109 | Ross LF, Clayton EW. Ethical Issues in Newborn Sequencing Research: The Case Study of BabySeq. Pediatrics. 2019;144(6). |
| 110 | Rothwell E, Botkin JR. Ethical Issues in Genetic Research with Infants: Biospecimen Use and Genome Sequencing. In: Kodish E, Nelson RM, editors. Ethics and research with children: A case-based approach (2nd ed). New York: Oxford University Press; 2019. p. 155-71. |
| 111 | Rotz SJ, Kodish E. Ethical conundrums in pediatric genomics. Hematology Am Soc Hematol Educ Program. 2018;2018(1):301-6. |
| 112 | Sabatello M, Appelbaum PS. Honey, I Sequenced the Kids: Preventive Genomics and the Complexities of Adolescence. The American journal of bioethics : AJOB. 2015;15(7):19-21. |
| 113 | Sabatello M, Appelbaum PS. Raising Genomic Citizens: Adolescents and the Return of Secondary Genomic Findings. J Law Med Ethics. 2016;44(2):292-308. |
| 114 | Sachdev R, Field M, Baynam GS, Beilby J, Berarducci M, Berman Y, et al. Paediatric genomic testing: Navigating medicare rebatable genomic testing. Journal of paediatrics and child health. 2021;57(4):477-83. |
| 115 | Sanderson SC, Lewis C, Patch C, Hill M, Bitner-Glindzicz M, Chitty LS. Opening the "black box" of informed consent appointments for genome sequencing: a multisite observational study. Genetics in medicine: official journal of the American College of Medical Genetics. 2019;21(5):1083-91. |
| 116 | Savatt JM, Wagner JK, Joffe S, Rahm AK, Williams MS, Bradbury AR, et al. Pediatric reporting of genomic results study (PROGRESS): a mixed-methods, longitudinal, observational cohort study protocol to explore disclosure of actionable adult- and pediatric-onset genomic variants to minors and their parents. BMC pediatrics. 2020;20(1):222. |
| 117 | Scollon S, Majumder MA, Bergstrom K, Wang T, McGuire AL, Robinson JO, et al. Exome sequencing disclosures in pediatric cancer care: Patterns of communication among oncologists, genetic counselors, and parents. Patient education and counseling. 2019;102(4):680-6. |
| 118 | Seidel MG. Baby genome screening: paving the way to genetic discrimination? Bmj. 2017;358:j3294. |
| 119 | Sénécal K, Rahimzadeh V, Knoppers BM, Fernandez CV, Avard D, Sinnett D. Statement of principles on the return of research results and incidental findings in paediatric research: a multi-site consultative process. Genome. 2015;58(12):541-8. |
| 120 | Smith EE, du Souich C, Dragojlovic N, Elliott AM. Genetic counseling considerations with rapid genome-wide sequencing in a neonatal intensive care unit. Journal of genetic counseling. 2019;28(2):263-72. |
| 121 | Smith LD, Willig LK, Kingsmore SF. Whole-Exome Sequencing and Whole-Genome Sequencing in Critically Ill Neonates Suspected to Have Single-Gene Disorders. Cold Spring Harb Perspect Med. 2015;6(2):a023168. |
| 122 | Sofer, D. The Fraught Reality of Genomic Sequencing. The American journal of nursing. 2020;120(3):14. |
| 123 | Sundby A, Boolsen MW, Burgdorf KS, Ullum H, Hansen TF, Mors O. Attitudes of stakeholders in psychiatry towards the inclusion of children in genomic research. Hum Genomics. 2018;12(1):12. |
| 124 | Szego MJ, Meyn MS, Shuman C, Zlotnik Shaul R, Anderson JA, Bowdin S, et al. Views from the clinic: Healthcare provider perspectives on whole genome sequencing in paediatrics. European journal of medical genetics. 2019;62(5):350-6. |
| 125 | Tarini BA, Goldenberg AJ. Ethical issues with newborn screening in the genomics era. Annu Rev Genomics Hum Genet. 2012;13:381-93. |
| 126 | Thornock BS. A strategic stakeholder approach for addressing further analysis requests in whole genome sequencing research. Life Sci Soc Policy. 2016;12:4. |
| 127 | Tibben A, Dondorp W, Cornelis C, Knoers N, Brilstra E, van Summeren M, et al. Parents, their children, whole exome sequencing and unsolicited findings: growing towards the child's future autonomy. European journal of human genetics: EJHG. 2021. |
| 128 | Uveges MK, Holm IA. Current Trends in Genetics and Neonatal Care. Advances in neonatal care: official journal of the National Association of Neonatal Nurses. 2021. |
| 129 | Vears DF. Should we respect parents' views about which results to return from genomic sequencing? Human genetics. 2021. |
| 130 | Vears DF, Borry P, Savulescu J, Koplin JJ. Old Challenges or New Issues? Genetic Health Professionals' Experiences Obtaining Informed Consent in Diagnostic Genomic Sequencing. AJOB empirical bioethics. 2021;12(1):12-23. |
| 131 | Vears DF, Sénécal K, Borry P. Genetic health professionals' experiences returning results from diagnostic genomic sequencing to patients. Journal of genetic counseling. 2020;29(5):807-15. |
| 132 | Vears DF, Sénécal K, Clarke AJ, Jackson L, Laberge AM, Lovrecic L, et al. Points to consider for laboratories reporting results from diagnostic genomic sequencing. Eur J Hum Genet. 2018;26(1):36-43. |
| 133 | Werner-Lin A, Zaspel L, Carlson M, Mueller R, Walser SA, Desai R, et al. Gratitude, protective buffering, and cognitive dissonance: How families respond to pediatric whole exome sequencing in the absence of actionable results. American journal of medical genetics Part A. 2018;176(3):578-88. |
| 134 | Wilfond B, Ross LF. From genetics to genomics: ethics, policy, and parental decision-making. J Pediatr Psychol. 2009;34(6):639-47. |
| 135 | Wilfond BS, Diekema DS. Engaging children in genomics research: decoding the meaning of assent in research. Genetics in medicine : official journal of the American College of Medical Genetics. 2012;14(4):437-43. |
| 136 | Wolf SM, Ossorio PN, Berry SA, Greely HT, McGuire AL, Penny MA, et al. Integrating Rules for Genomic Research, Clinical Care, Public Health Screening and DTC Testing: Creating Translational Law for Translational Genomics. The Journal of law, medicine & ethics: a journal of the American Society of Law, Medicine & Ethics. 2020;48(1):69-86. |
| 137 | Wong CS, Kogon AJ, Warady BA, Furth SL, Lantos JD, Wilfond BS. Ethical and Policy Considerations for Genomic Testing in Pediatric Research: The Path Toward Disclosing Individual Research Results. American journal of kidney diseases : the official journal of the National Kidney Foundation. 2019;73(6):837-45. |
| 138 | Woudstra A, Dondorp W, de Wert G. Stakeholder views on opportunistic genomic screening in the Netherlands: a qualitative study. European journal of human genetics : EJHG. 2021. |
| 139 | Wouters RHP, Cornelis C, Newson AJ, Bunnik EM, Bredenoord AL. Scanning the body, sequencing the genome: Dealing with unsolicited findings. Bioethics. 2017;31(9):648-56. |
| 140 | Yang L, Chen J, Shen B. Newborn Screening in the Era of Precision Medicine. Adv Exp Med Biol. 2017;1005:47-61. |
| 141 | Yu JH, Appelbaum PS, Brothers KB, Joffe S, Kauffman TL, Koenig BA, et al. Consent for clinical genome sequencing: considerations from the Clinical Sequencing Exploratory Research Consortium. Personalized medicine. 2019;16(4):325-33. |
| 142 | Zacharias RL, Smith ME, King JS. The Legal Dimensions of Genomic Sequencing in Newborn Screening. The Hastings Center report. 2018;48 Suppl 2:S39-s41. |
| 143 | Zawati MH, Parry D, Knoppers BM. The best interests of the child and the return of results in genetic research: international comparative perspectives. BMC Med Ethics. 2014;15:72. |
